# Sulfation Rewrites the Biological Profile of Dietary Flavonoids: Implications for Stability, Redox Signaling, and Inflammation

**DOI:** 10.1002/fsn3.72382

**Published:** 2026-09-21

**Authors:** Jiří Vrba, Veronika Zapletalová, Jakub Havlásek, Barbora Papoušková, Barbora Petránková, Helena Pelantová, Kateřina Valentová

**Affiliations:** ^1^ Department of Medical Chemistry and Biochemistry, Faculty of Medicine and Dentistry Palacký University Olomouc Czechia; ^2^ Department of Analytical Chemistry, Faculty of Science Palacký University Olomouc Czechia; ^3^ Institute of Microbiology of the Czech Academy of Sciences, Laboratory of Biotransformation Prague Czechia; ^4^ Department of Genetics and Microbiology, Faculty of Science Charles University Prague Czechia; ^5^ Institute of Microbiology of the Czech Academy of Sciences Laboratory of Molecular Structure Characterization Prague Czechia

**Keywords:** flavonoid, metabolism, NF‐kappaB, NRF2, sulfate

## Abstract

Dietary flavonoids undergo extensive Phase II metabolism, yet the functional consequences of sulfation for their biological activity remain poorly understood. In this study, we compared the physicochemical properties and cellular effects of nine structurally diverse flavonoids and their sulfated derivatives. Flavonoid 3′‐*O*‐ or 4′‐*O*‐sulfates were prepared enzymatically using bacterial aryl sulfotransferase, structurally confirmed, and evaluated as potential metabolites that can be formed by human cytosolic sulfotransferases (SULTs). The results showed that sulfation significantly increased the stability of otherwise labile flavonoids under physiologically relevant conditions, while generally reducing their antiradical capacity and cytotoxicity toward murine RAW264.7 macrophage cells. In contrast to nonsulfated flavonoids, particularly fisetin and quercetin, all tested flavonoid sulfates failed to activate the NRF2 (nuclear factor erythroid 2 p45‐related factor 2) pathway in RAW264.7 cells. On the other hand, some of the parent flavonoids as well as flavonoid sulfates partially attenuated lipopolysaccharide‐stimulated expression of cyclooxygenase‐2 (COX‐2) and inducible nitric oxide synthase (iNOS), indicating that sulfated metabolites retain modulatory effects on inflammatory responses. Using recombinant human sulfotransferases, we identified multiple enzymes responsible for the formation of the tested sulfates, confirming their relevance as plausible metabolites. Overall, our findings demonstrate that metabolic sulfation profoundly reshapes flavonoid stability and biological activity, underscoring the importance of considering conjugated metabolites when evaluating the health effects of dietary flavonoids.

## Graphical abstract

Enzymatic sulfation of dietary flavonoids increases their stability while attenuating antioxidant activity, cytotoxicity, and NRF2 activation. Sulfated flavonoids remain biologically active, modulating inflammatory markers, and represent authentic human metabolites relevant for nutritional and metabolic studies.

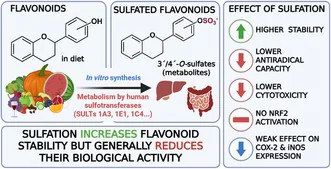

**Abbreviations**
AM: ampelopsin
AM‐S: ampelopsin‐4′‐*O*‐sulfate
ANOVA: analysis of variance
AP: apigenin
AP‐S: apigenin‐4′‐*O*‐sulfate
COX‐2/PTGS2: cyclooxygenase‐2
*dh*AST: aryl sulfotransferase from *Desulfitobacterium hafniense*

DMSO: dimethyl sulfoxide
DPPH: 1,1‐diphenyl‐2‐picrylhydrazyl
EC_50_
: half‐maximal effective concentration
FI: fisetin
FI‐S: fisetin‐3′‐*O*‐sulfate
GAPDH: glyceraldehyde‐3‐phosphate dehydrogenase
GE: genistein
GE‐S: genistein‐4′‐*O*‐sulfate
HE: hesperetin
HE‐S: hesperetin‐3′‐*O*‐sulfate
HMBC: heteronuclear multiple bond correlation
HO‐1/HMOX1: heme oxygenase‐1
HPLC: high‐performance liquid chromatography
iNOS/NOS2: inducible nitric oxide synthase
KEAP1: Kelch‐like ECH‐associated protein 1
LC: liquid chromatography
LPS: lipopolysaccharide
MS: mass spectrometry
MTT: 3‐(4,5‐dimethylthiazol‐2‐yl)‐2,5‐diphenyltetrazolium bromide
MY: myricetin
MY‐S: myricetin‐3′‐*O*‐sulfate
NA: naringenin
NA‐S: naringenin‐4′‐*O*‐sulfate
NF‐κB: nuclear factor‐κB
NMR: nuclear magnetic resonance
NQO1: NAD(P)H:quinone oxidoreductase 1
NRF2/NFE2L2: nuclear factor erythroid 2 p45‐related factor 2
PCR: polymerase chain reaction
QUE: quercetin
QUE‐S: quercetin‐3′‐*O*‐sulfate
SAR/SPAR: structure–activity/structure–property‐activity relationship
SD: standard deviation
SULT: sulfotransferase
TAX: taxifolin
TAX‐S: taxifolin‐4′‐*O*‐sulfate

## 1 Introduction

**FIGURE 1:**
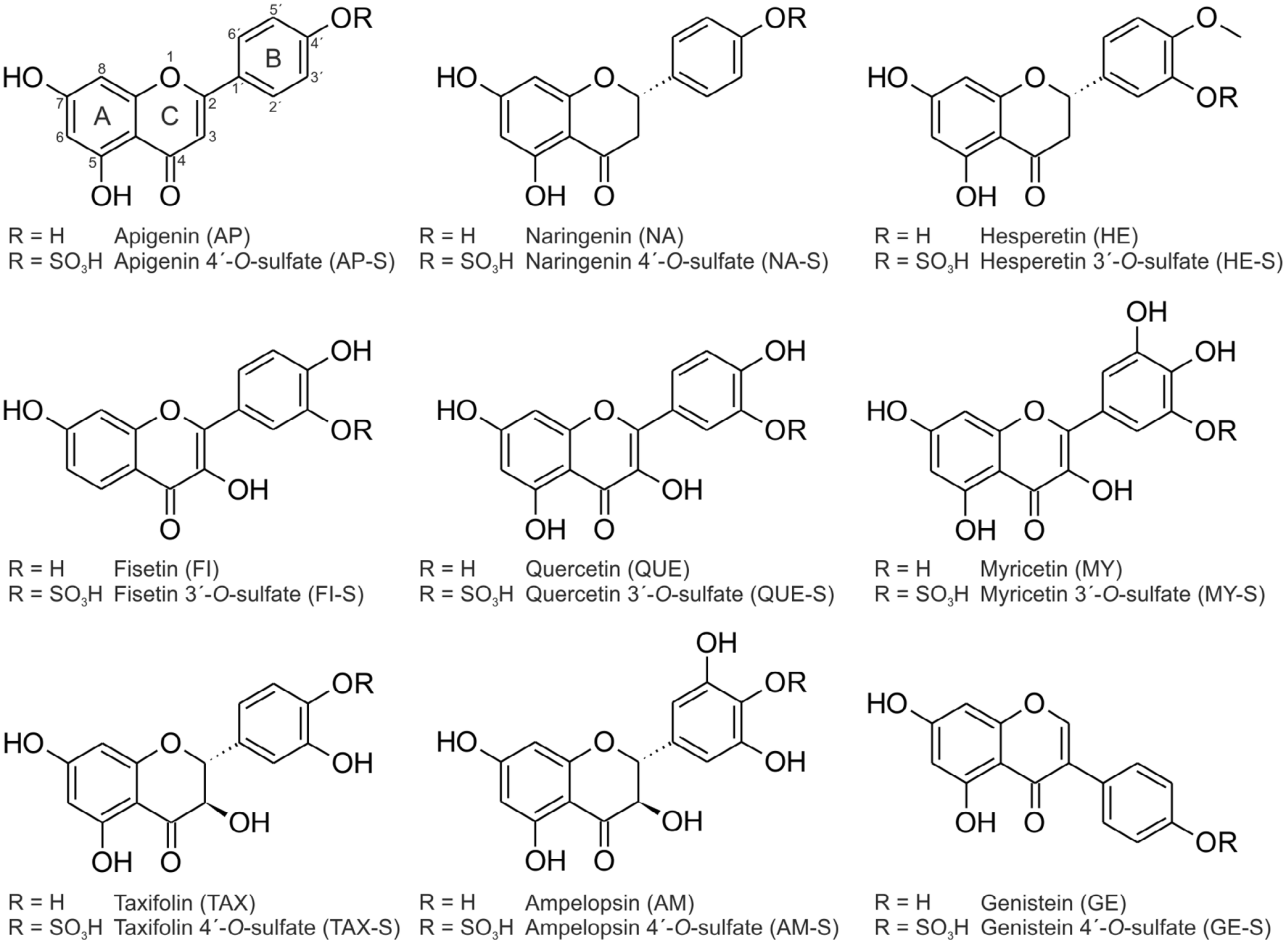
Tested flavonoids and their sulfates.

Natural flavonoids constitute a large and structurally diverse group of plant secondary metabolites composed of two benzene rings (A and B) linked by a heterocyclic C ring (Figure 1). According to the position of ring B and the oxidation state of the core scaffold, flavonoids are classified into subclasses such as flavones, isoflavones, and flavanones. Their structural diversity is further increased by modifications of hydroxyl groups, most commonly glycosylation or methylation (Taniguchi et al. 2023). In contrast, sulfation of flavonoids is relatively rare in plants and has been mainly associated with species growing in wetland or aquatic environments (Teles et al. 2018). From a nutritional perspective, flavonoid‐rich diets have been consistently associated with a reduced risk of cardiovascular diseases (Williamson 2017). However, understanding the molecular basis of these health benefits requires moving beyond the properties of parent compounds found in food, as dietary flavonoids undergo extensive metabolic transformation after ingestion.

At the molecular level, the beneficial effects of flavonoids are partly attributed to modulation of cellular signaling pathways. Several flavonoids, including quercetin, naringenin, and genistein, activate the transcription factor nuclear factor erythroid 2 p45‐related factor 2 (NRF2 or NFE2L2), a key regulator of cytoprotective and antioxidant responses (Suraweera et al. 2020). Under normal conditions, NRF2 interacts with its negative regulatory protein Kelch‐like ECH‐associated protein 1 (KEAP1), which keeps NRF2 inactive in the cytoplasm and promotes its degradation in the proteasome. Under stress conditions, NRF2 dissociates from its inhibitor KEAP1, translocates to the nucleus, and induces genes such as those encoding heme oxygenase‐1 (HO‐1 or HMOX1) and NAD(P)H:quinone oxidoreductase 1 (NQO1) (Dinkova‐Kostova et al. 2024; Liu et al. 2021). In addition, flavonoids may exert anti‐inflammatory effects by suppressing activation of the nuclear factor‐κB (NF‐κB) pathway, which regulates the expression of pro‐inflammatory genes including cyclooxygenase‐2 (COX‐2 or PTGS2) and inducible nitric oxide synthase (iNOS or NOS2) (Giridharan and Srinivasan 2018; Pandey et al. 2024).

In humans, dietary flavonoids undergo extensive metabolism by gut microbiota and host tissues, particularly in the intestine and liver. Following deglycosylation, aglycones are rapidly conjugated via Phase II reactions, including sulfation, glucuronidation, and methylation (Wang et al. 2023). As a result, circulating levels of parent flavonoids are exceedingly low, typically in the nanomolar range even after flavonoid‐rich meals. In contrast, conjugated metabolites, particularly glucuronides and sulfates, reach substantially higher concentrations and persist in plasma for several hours. For instance, a recent pharmacokinetic study in humans demonstrated that, after oral intake of quercetin, monosulfates, monoglucuronides, and, notably, hetero‐conjugates bearing both glucuronic acid and sulfate moieties are major circulating species (Sudaka et al. 2025). Consequently, conclusions about the biological effects of dietary flavonoids based solely on the parent aglycones may misinterpret their nutritional and functional significance for humans.

Although glucuronides are generally considered to be the main type of xenobiotic metabolites, several considerations have motivated us to focus on flavonoid sulfates: (a) tissues with a sufficient expression of sulfotransferases (SULTs), e.g., intestinal epithelium (Coughtrie 2016; Riches et al. 2009), may produce relevant amounts of flavonoid sulfates; (b) flavonoid sulfation may occur at distinct hydroxyl groups than glucuronidation, thus conferring different biological activities compared to glucuronides; (c) emerging evidence suggests that flavonoid sulfates can serve as reservoirs for bioactive aglycones through the action of sulfatases at sites of inflammation (Oliveira et al. 2026); and (d) the lack of information on the bioactivity of individual flavonoid sulfates represents a critical knowledge gap in our understanding the flavonoid metabolism–activity relationships.

The present study therefore examined the impact of flavonoid sulfation on physicochemical properties and biological activities, including antioxidant capacity, cytotoxicity, and interactions with the NRF2 and NF‐κB pathways. Nine flavonoids and their corresponding monosulfates were investigated, four of which were synthesized for the first time using aryl sulfotransferase from *Desulfitobacterium hafniense* (*dh*AST) (Brodsky et al. 2026). In parallel, human sulfotransferases responsible for flavonoid sulfation were identified.

## 2 Materials and Methods

### 2.1 The Parent Flavonoids

Ampelopsin (94% purity) was obtained from Herb Nutritionals (Shanghai, China), apigenin (97%) and hesperetin (99.5%) were from Biosynth (Compton, UK), fisetin (96%) was from Thermo Fisher Scientific (Heysham, UK), myricetin (97%) was from ABCR (Karlsruhe, Germany), genistein (100%), naringenin (98%), and quercetin (98%) were from Merck/Sigma‐Aldrich (Darmstadt, Germany), and taxifolin (96%) was from Amagro (Prague, Czechia).

### 2.2 Flavonoid Sulfates

Flavonoid sulfates were prepared by the sulfation of respective parent compounds using recombinant *dh*AST, expressed in *Escherichia coli* , and *p*‐nitrophenyl sulfate (*p*‐NPS) as a sulfate donor. Details on the enzymatic preparation of ampelopsin‐4′‐*O*‐sulfate (96%) and myricetin‐3′‐*O*‐sulfate (99%) have been reported previously (Káňová et al. 2020), as have those for naringenin‐4′‐*O*‐sulfate (100%) (Kaci et al. 2023), quercetin‐3′‐*O*‐sulfate (98%) and taxifolin‐4′‐*O*‐sulfate (97%) in (Purchartová et al. 2015; Roubalova et al. 2015).

Enzymatic sulfation using *dh*AST and *p*‐NPS was further applied to generate the novel flavonoid sulfates apigenin‐4′‐*O*‐sulfate, fisetin‐3′‐*O*‐sulfate, genistein‐4′‐*O*‐sulfate, and hesperetin‐3′‐*O*‐sulfate; experimental details are provided in the Supporting Information (page 3–4, Table S1).

### 2.3 Absorption Spectroscopy of Tested Flavonoids

Flavonoids were freshly dissolved in methanol, ultra‐LC–MS grade (VWR International, Stříbrná Skalice, Czechia), to a concentration of 1 mM, then diluted with methanol to 10 μM. The absorption spectra of 10 μM flavonoid solutions were recorded at room temperature (rt) from 220 to 450 nm against pure methanol using 1‐cm quartz cuvettes in a UV‐2700i UV–Vis spectrophotometer (Shimadzu, Kyoto, Japan).

### 2.4 Spectrophotometric Evaluation of Flavonoid Stability

Solutions of 1 mM flavonoids in methanol were diluted with 50 mM sodium phosphate buffer, pH 7.4, to 10 μM, and incubated for 0–24 h at 37°C and 300 rpm in a Comfort Thermomixer (Eppendorf, Hamburg, Germany). After incubation, absorption spectra were recorded at rt. from 220 to 450 nm against phosphate buffer containing 1% (*v/v*) methanol using 1‐cm quartz cuvettes in a UV‐2700i UV–Vis spectrophotometer (Shimadzu). Absorbance values at the appropriate absorption maxima were used to calculate the relative concentrations of flavonoids, with absorbance values at 0 h representing 100%.

### 2.5 DPPH Radical Scavenging Assay

The ability of the tested flavonoids to scavenge 1,1‐diphenyl‐2‐picrylhydrazyl (DPPH) radicals was determined at final flavonoid concentrations of 0–50 μM as described previously (Roubalova et al. 2015).

### 2.6 Cell Culture and Treatments

RAW264.7 murine macrophage cells (ECACC, Salisbury, UK) were cultured and seeded for experiments as described previously (Roubalova et al. 2015). After seeding, the cells were incubated overnight and then treated as described below.

To examine the effect of sulfation on possible cytotoxicity of the tested flavonoids, RAW264.7 cells were treated for 24 h in serum‐free medium with 0.1% (*v/v*) dimethyl sulfoxide (DMSO, negative control), 1–50 μM flavonoids, or 1.5% (*v/v*) Triton X‐100 (positive control).

To test possible activation of the Nrf2 pathway, RAW264.7 cells were treated for 4 h in serum‐free medium with 0.1% (*v/v*) DMSO (negative control), 50 μM flavonoids, or 5 μM sulforaphane (positive control).

To evaluate the effect of the tested compounds on LPS‐induced inflammatory response, RAW264.7 cells were pretreated for 2 h in serum‐containing medium with 0.1% (*v/v*) DMSO (negative control), 10 μM flavonoids, or 1 μM dexamethasone (positive control). The cells were then co‐treated for an additional 4 h (for gene expression analysis) or 22 h (for protein expression analysis) with the tested compounds or DMSO and 0.1 μg/mL lipopolysaccharide (LPS) from *E. coli* O55:B5 (L4524, Merck/Sigma‐Aldrich). An additional negative control was incubated with DMSO in the absence of LPS.

### 2.7 Cell Viability Assay

After the treatments described in Section 2.6, the viability of RAW264.7 cells was determined using the 3‐(4,5‐dimethylthiazol‐2‐yl)‐2,5‐diphenyltetrazolium bromide (MTT) reduction assay as described previously (Roubalova et al. 2015).

### 2.8 Quantitative Real‐Time PCR

RAW264.7 cells were treated with the tested compounds as described in Section 2.6, and polymerase chain reaction (PCR) analysis of relative gene expression was performed (Roubalova et al. 2015). For amplification, TaqMan gene expression assays (Life Technologies/Applied Biosystems, Grand Island, NY, USA) with the following assay IDs were used: Mm99999915_g1 (glyceraldehyde‐3‐phosphate dehydrogenase, Gapdh), Mm00516005_m1 (Hmox1), Mm01253561_m1 (Nqo1), Mm00477784_m1 (Nfe2l2, alias Nrf2), Mm00478374_m1 (Ptgs2, alias Cox2), and Mm00440502_m1 (Nos2). Relative changes in gene expression were calculated by the comparative *C*_T_ method using the equation 2^−ΔΔCt^, and the results were normalized to Gapdh mRNA levels.

### 2.9 Western Blot Analysis

RAW264.7 cells were treated as described in Section 2.6, and Western blot analysis of protein expression was performed (Roubalova et al. 2015). Electrophoresis was conducted using 4%–15% TGX precast protein gels (Bio‐Rad Laboratories, Hercules, CA, USA). Rabbit monoclonal NRF2 antibody (ab62352) and rabbit monoclonal KEAP1 antibody (ab227828) were obtained from Abcam (Cambridge, MA, USA). Rabbit polyclonal HO‐1 antibody (No. 70081), rabbit monoclonal COX2 (D5H5) XP antibody (No. 12282), rabbit monoclonal iNOS (E1W4J) antibody (No. 68186), rabbit monoclonal GAPDH (D16H11) XP antibody (No. 5174), rabbit monoclonal β‐actin (D6A8) antibody (No. 8457), and goat anti‐rabbit IgG‐HRP antibody (No. 7074) were obtained from Cell Signaling Technology (Danvers, MA, USA). Chemiluminescence produced by a Western blotting luminol reagent (Santa Cruz Biotechnology, Santa Cruz, CA, USA) was imaged using a G:Box Chemi‐XX6 system (Syngene, Cambridge, UK), and relative band intensity was analyzed using ImageJ software (National Institutes of Health, Bethesda, MD, USA).

### 2.10 Sulfation of Flavonoids by Human Sulfotransferases (SULTs) and LC–MS Analysis

Cytosolic fractions from *E. coli* expressing recombinant human SULT1A1*1, SULT1A1*2, SULT1A2, SULT1A3, SULT1B1, SULT1C2, SULT1C4, SULT1E1, and SULT2A1 were obtained from Cypex (Dundee, UK). The tested flavonoids (50 μM in 0.1% (*v/v*) DMSO) were incubated for 30 min at 37°C without agitation in 50 mM potassium phosphate–HCl buffer (pH 7.4) containing 10 mM dithiothreitol, 5 mM MgCl_2_, 50 μg/mL *E. coli* cytosol protein, and 120 μM 3′‐phosphoadenosine 5′‐phosphosulfate (PAPS; Merck/Sigma‐Aldrich). Spiked samples were prepared by adding the tested flavonoid sulfates (10 μM) to the incubation mixtures at the end of incubation. Control samples were prepared without PAPS (cofactor) and/or the cytosolic fractions (enzymes). Incubations were performed in a Comfort Thermomixer (Eppendorf). Samples were then stored at −80°C and analyzed by liquid chromatography‐mass spectrometry (LC–MS) as described previously (Vrba et al. 2021) with some differences in LC separation. For details, see the Supporting Information.

### 2.11 Dosage Information

All experiments were conducted in vitro. Flavonoids and their sulfates were applied to the culture medium at concentrations of 1–50 μM from DMSO stock solutions, with a final solvent concentration not exceeding 0.1% (*v/v*). Cytotoxicity was assessed after 24 h of exposure, NRF2 pathway activation after 4 h treatment with 50 μM flavonoids, and anti‐inflammatory effects after 2 h pretreatment with 10 μM flavonoids followed by cotreatment with LPS for 4 and 22 h.

The concentration range was selected based on previous mechanistic in vitro studies on flavonoids (Chuang et al. 2017; Ren et al. 2016; Roubalova et al. 2016; Tanigawa et al. 2007; Vrba et al. 2013; Zhang et al. 2019; Zhang et al. 2022). Cell viability remained above 85% at the applied concentrations (Figure S17). Although concentrations above 10 μM exceed plasma levels achievable through regular diet, they are commonly used in cell‐based studies to account for limited cellular uptake and rapid metabolism of flavonoids (Káňová et al. 2020; Roubalova et al. 2015). Notably, total flavonoid conjugate concentrations approaching 10 μM have been reported in human plasma after ingestion of appropriately formulated quercetin supplements. For example, an isoquercitrin‐γ‐cyclodextrin inclusion complex increased the maximum plasma concentration of total quercetin conjugates to 9.4 μM in healthy volunteers (Williamson 2025). The concentration of 10 μM, used for the anti‐inflammatory experiments, is therefore within the upper range achievable after dietary supplementation, while the 50 μM concentration used for the NRF2 pathway experiments was selected to establish maximal activation capacity and to account for the short exposure time (4 h) relative to chronic dietary intake. The relevance of sulfated flavonoid metabolites at these concentrations is further supported by recent pharmacokinetic data showing that quercetin monosulfates belong among the predominant circulating species in human plasma after flavonoid ingestion (Sudaka et al. 2025). In addition, since the sulfation reduces passive membrane permeability, the intracellular concentrations of flavonoid sulfates were likely lower than those of the parent aglycones at the same nominal medium concentrations. The cellular effects of sulfate flavonoids reported here should therefore be interpreted as conservative estimates of their potency.

### 2.12 Statistical Analysis

Results were expressed as means ± standard deviation (SD). Statistical analysis was performed using Statistica software (TIBCO Software, Palo Alto, CA, USA). One‐way analysis of variance (ANOVA) followed by Dunnett's test was used for comparison of data with respective control values, and two‐way ANOVA followed by Tukey's test was used for comparison between data for sulfated and nonsulfated compounds. A *p* value of less than 0.05 was considered to be statistically significant.

## 3 Results and Discussion

### 3.1 Synthesis of Flavonoid Sulfates

The study compared selected physicochemical and biological properties of nine flavonoids representing major flavonoid classes (apigenin, naringenin, hesperetin, fisetin, quercetin, myricetin, taxifolin, ampelopsin, and genistein) and their corresponding monosulfates. Sulfation occurred at the 3′‐OH or 4′‐OH position of ring B. Naringenin‐4′‐*O*‐sulfate, quercetin‐3′‐*O*‐sulfate, myricetin‐3′‐*O*‐sulfate, taxifolin‐4′‐*O*‐sulfate, and ampelopsin‐4′‐*O*‐sulfate were synthesized using recombinant *dh*AST as previously reported (Kaci et al. 2023; Káňová et al. 2020; Purchartová et al. 2015; Roubalova et al. 2015). The same approach was applied to apigenin, fisetin, genistein, and hesperetin, yielding apigenin‐4′‐*O*‐sulfate, fisetin‐3′‐*O*‐sulfate, genistein‐4′‐*O*‐sulfate, and hesperetin‐3′‐*O*‐sulfate. Isolated yields ranged from 8% to 29%, with purities of 96%–100% (Table 1). Sulfation was confirmed by mass spectrometry (MS), while structures were assigned by ^1^H and ^13^C nuclear magnetic resonance (NMR, Table 2; Tables S2–S4; Figures S1–S16). As diagnostic heteronuclear multiple bond correlation (HMBC) correlations were not observable due to broad hydroxyl signals, sulfate positions were determined indirectly from characteristic changes in carbon chemical shifts relative to the parent flavonoids, consistent with reported catechol sulfation patterns (Purchartová et al. 2015).

**TABLE 1:** Characteristics of newly prepared flavonoid sulfates and isolated yields of sulfation.

Compound	Purity^a^	m/z	Retention time^b^	Isolated yield		HPLC^b^	NMR^b^	MS^b^
	(%)	[M – H]^−^	(min)	(mg/100 mg of starting material)	(mol%)			
Apigenin			20.925					
Apigenin‐4′‐*O*‐sulfate	96	349	13.979	10.3	8	Figure S1	Figures S2 and S3; Table S2	Figure S4
Fisetin			13.273					
Fisetin‐3′‐*O*‐sulfate	100	365	11.915	28.2	22	Figure S5	Figures S6 and S7; Table S3	Figure S8
Genistein			16.080					
Genistein‐4′‐*O*‐sulfate	100	349	11.915	25	19	Figure S9	Figures S10 and S11; Table S4	Figure S12
Hesperetin			18.462					
Hesperetin‐3′‐*O*‐sulfate	100	381	10.641	36	29	Figure S13	Figures S14 and S15; Table S5	Figure S16

^a^ Determined by HPLC.

^b^ Details in Supporting Information.

**TABLE 2:** Absorption spectral properties of flavonoids in methanol.

Compound	CAS	*λ* in nm (*ε* in M^−1^ cm^−1^)
*I Flavones*		
Apigenin	520‐36‐5	267 (20300), 334 (23050)
Apigenin‐4′‐*O*‐sulfate		269 (26350), 317 (17650)
*II Flavanones*		
Naringenin	480‐41‐1	287 (21650)
Naringenin‐4′‐*O*‐sulfate		288 (16800)
Hesperetin	520‐33‐2	287 (17100)
Hesperetin‐3′‐*O*‐sulfate		287 (12050)
*III Flavonols*		
Fisetin	528‐48‐3	248 (20050), 318 (14500), 362 (28950)
Fisetin‐3′‐*O*‐sulfate		321 (8100), 355 (12350)
Quercetin	117‐39‐5	255 (26200), 302 (10600), 371 (27200)
Quercetin‐3′‐*O*‐sulfate	62369‐28‐2	249 (13150), 266 (13750), 322 (9300), 366 (17100)
Myricetin	529‐44‐2	253 (22450), 305 (8250), 375 (27900)
Myricetin‐3′‐*O*‐sulfate		253 (15250), 371 (15850)
*IV Flavanonols*		
Taxifolin	480‐18‐2	288 (16800)
Taxifolin‐4′‐*O*‐sulfate		289 (15850)
Ampelopsin	27200‐12‐0	291 (20200)
Ampelopsin‐4′‐*O*‐sulfate		290 (20500)
*V Isoflavones*		
Genistein	446‐72‐0	261 (31800)
Genistein‐4′‐*O*‐sulfate		259 (25200)

*Note:* From absorption spectra of 10 μM flavonoids in methanol, at rt. and 220–450 nm. Wavelengths of absorption maxima (*λ*) were determined with an accuracy of 1 nm, and molar absorption coefficients (*ε*) were calculated according to Lambert–Beer's law.

### 3.2 Absorption Spectra of Tested Flavonoids

**FIGURE 2:**
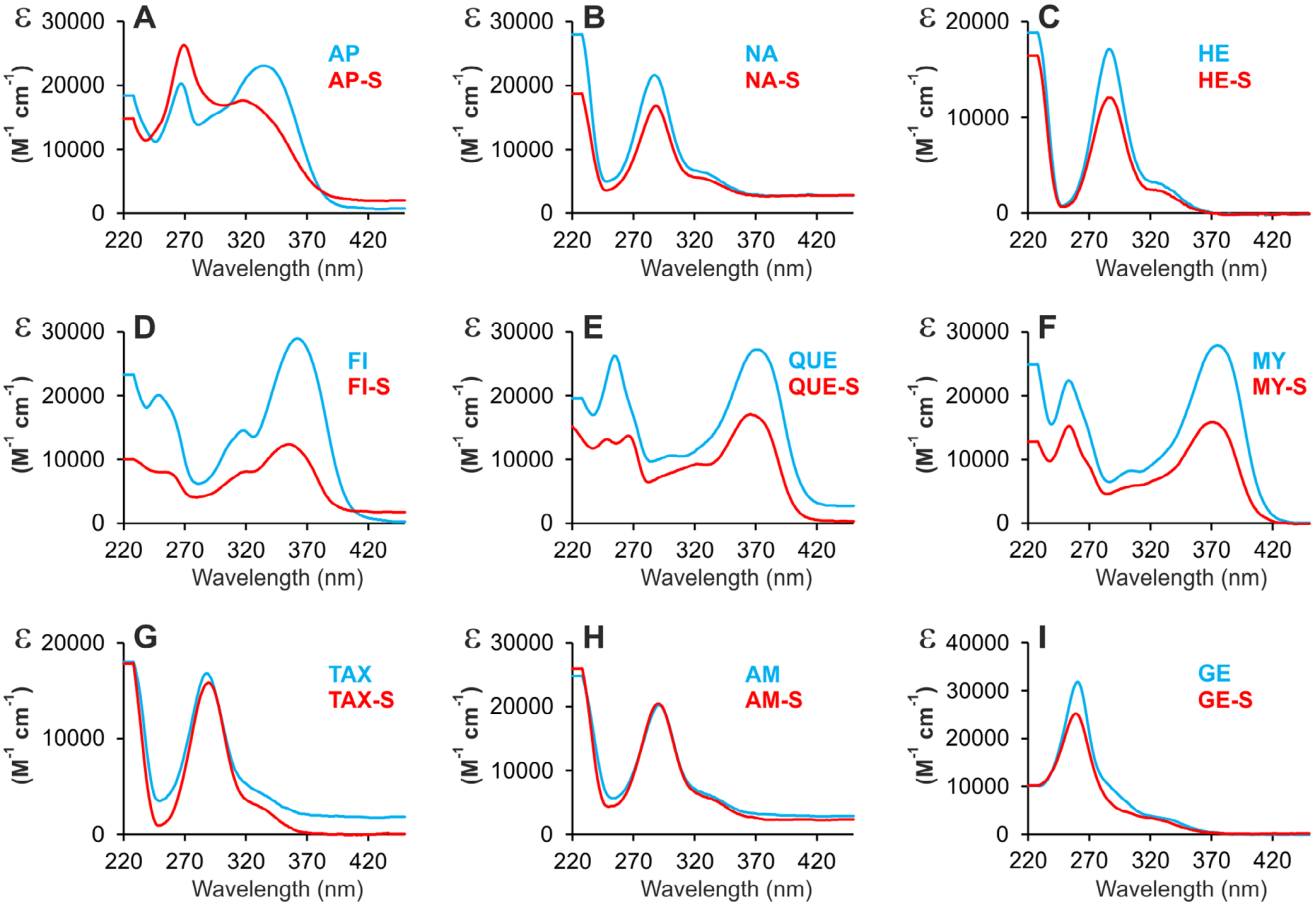
Absorption spectra of tested flavonoids (blue) and their sulfates (red) in methanol. (A) Apigenin; (B) naringenin; (C) hesperetin; (D) fisetin; (E) quercetin; (F) myricetin; (G) taxifolin; (H) ampelopsin; (I) genistein. Absorption maxima (λ) and corresponding molar absorption coefficients (*ε*) can be found in Table 3.

Flavonoids containing a 2,3‐double bond and ring B at C2 typically display two UV–vis absorption maxima: band II (240–295 nm), associated with the benzoyl system of rings A–C, and band I (300–400 nm), corresponding to the cinnamoyl system of rings B–C (Halbwirth 2010; Taniguchi et al. 2023). Accordingly, apigenin and the flavonols fisetin, quercetin, and myricetin exhibited two main maxima in methanol, with flavonols additionally showing a weaker intermediate maximum at 302–318 nm (Figure 2 and Table S6). In contrast, flavanones (naringenin, hesperetin), flavanonols (taxifolin, ampelopsin), which lack the 2,3‐double bond, and the isoflavone genistein, with ring B at C3, showed a single maximum corresponding to band II.

Sulfation altered the absorption characteristics of several flavonoids. Apigenin‐4′‐*O*‐sulfate displayed decreased absorption in band I and increased absorption in band II relative to apigenin. For fisetin, quercetin, and myricetin, 3′‐*O*‐sulfation markedly reduced absorption across the spectrum and induced changes in band II features, including peak splitting or disappearance. Monosulfates of naringenin, hesperetin, and genistein showed lower absorption than their parent compounds, whereas taxifolin‐4′‐*O*‐sulfate and ampelopsin‐4′‐*O*‐sulfate exhibited spectra comparable to the corresponding aglycones. Sulfation also caused shifts in absorption maxima, most pronounced for apigenin and quercetin. Absorption maxima and molar absorption coefficients are summarized in Table 2, with nonsulfated flavonoids consistent with published data (Taniguchi et al. 2023).

### 3.3 Stability of Tested Flavonoids

**FIGURE 3:**
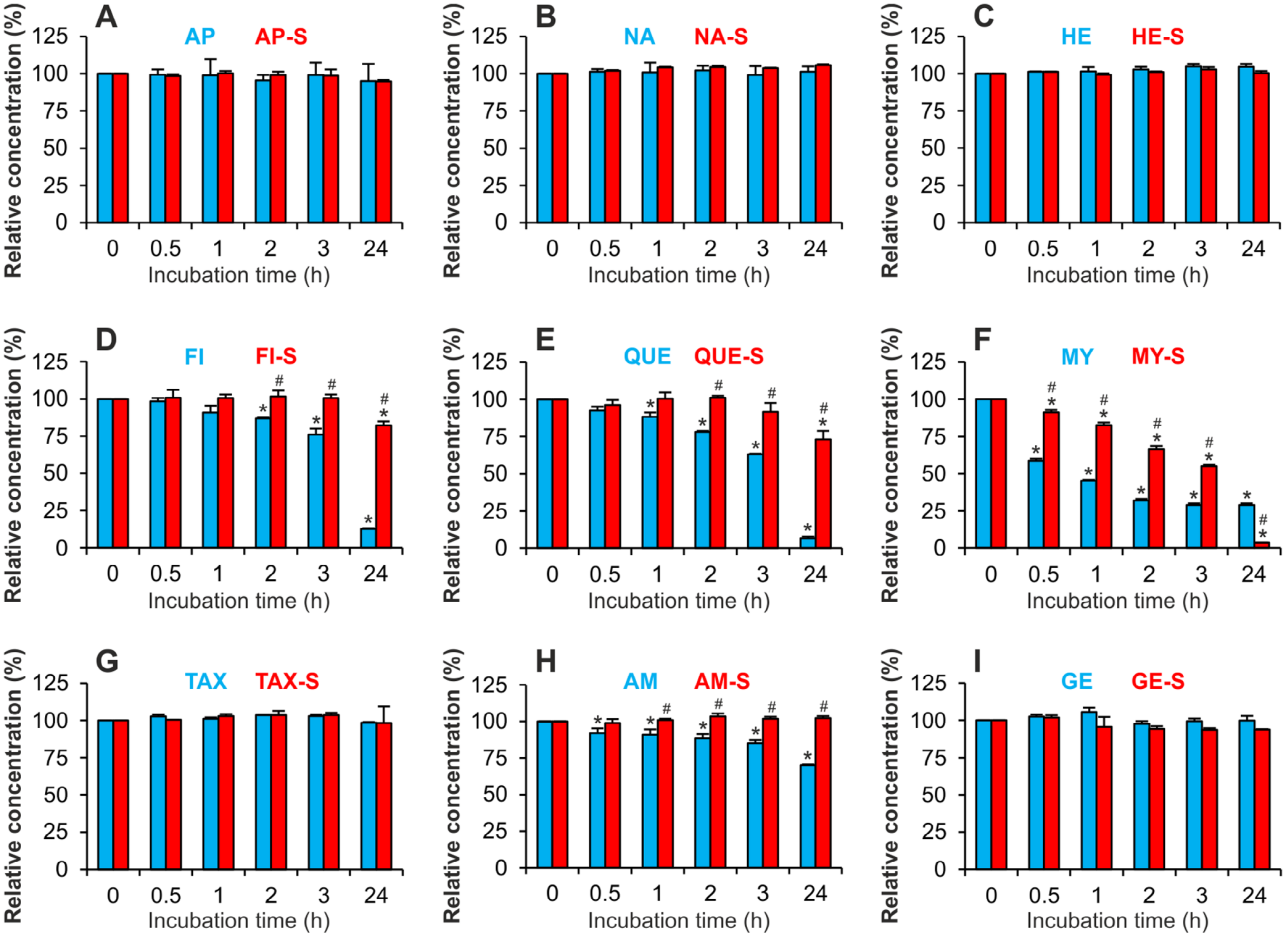
Stability of tested flavonoids (blue) and their sulfates (red). Flavonoids (10 μM) were incubated in 50 mM sodium phosphate buffer (pH 7.4) for 24 h at 37°C, and at the indicated time points, their relative concentrations were determined from their absorption spectra at appropriate absorption maxima (wavelengths quoted in brackets): (A) apigenin (270 nm) and its 4′‐*O*‐sulfate (271 nm); (B) naringenin (322 nm) and its 4′‐*O*‐sulfate (322 nm); (C) hesperetin (322 nm) and its 3′‐*O*‐sulfate (322 nm); (D) fisetin and its 3′‐*O*‐sulfate (362 nm); (E) quercetin (378 nm) and its 3′‐*O*‐sulfate (374 nm); (F) myricetin (377 nm) and its 3′‐*O*‐sulfate (378 nm); (G) taxifolin (324 nm) and its 4′‐*O*‐sulfate (325 nm); (H) ampelopsin (325 nm) and its 4′‐*O*‐sulfate (325 nm); (I) genistein (264 nm) and its 4′‐*O*‐sulfate (266 nm). Data are means ± SD of three experiments. *p < 0.05, significantly decreased versus the control value (i.e., at the time point of 0 h). #*p* < 0.05, significantly different from the value for the respective nonsulfated flavonoid. Absorption maxima (*λ*) and corresponding molar absorption coefficients (*ε*) at 0 h can be found in Table S5.

The stability of the tested compounds (10 μM) was assessed spectrophotometrically under cell culture–relevant conditions (phosphate buffer, pH 7.4, 37°C). After 24 h, apigenin, naringenin, hesperetin, taxifolin, genistein, and their sulfates remained stable, retaining > 94% of their initial concentrations (Figure 3). In contrast, the remaining parent flavonoids were unstable, with decreasing stability in the order ampelopsin > fisetin > quercetin > myricetin, consistent with previous reports (Xiao and Hogger 2015). After 3 h, concentrations of myricetin, quercetin, fisetin, and ampelopsin declined to 29%, 63%, 76%, and 85%, respectively, indicating that flavonols are the most labile subclass and that instability correlates with the number of hydroxyl groups. Myricetin showed rapid degradation, dropping to 59% within 30 min, although its absorbance after 24 h declined less than that of quercetin and fisetin.

Sulfation significantly improved the stability of these labile flavonoids. After 3 h, myricetin‐3′‐*O*‐sulfate and quercetin‐3′‐*O*‐sulfate retained 55% and 92% of their initial concentrations, respectively, while fisetin‐3′‐*O*‐sulfate and ampelopsin‐4′‐*O*‐sulfate showed no loss. Myricetin‐3′‐*O*‐sulfate was the least stable sulfate, decreasing to 4% after 24 h, whereas enhanced stability of sulfated fisetin, quercetin, and ampelopsin became more evident at longer incubation times (Figure 3). These data demonstrate that sulfation of a single hydroxyl group can markedly affect flavonoid stability, similar to the stabilizing effect previously reported for glycosylation (Xiao and Hogger 2015). Absorption maxima and molar absorption coefficients in phosphate buffer are summarized in Table S6.

### 3.4 Antiradical Activity of Tested Flavonoids

**FIGURE 4:**
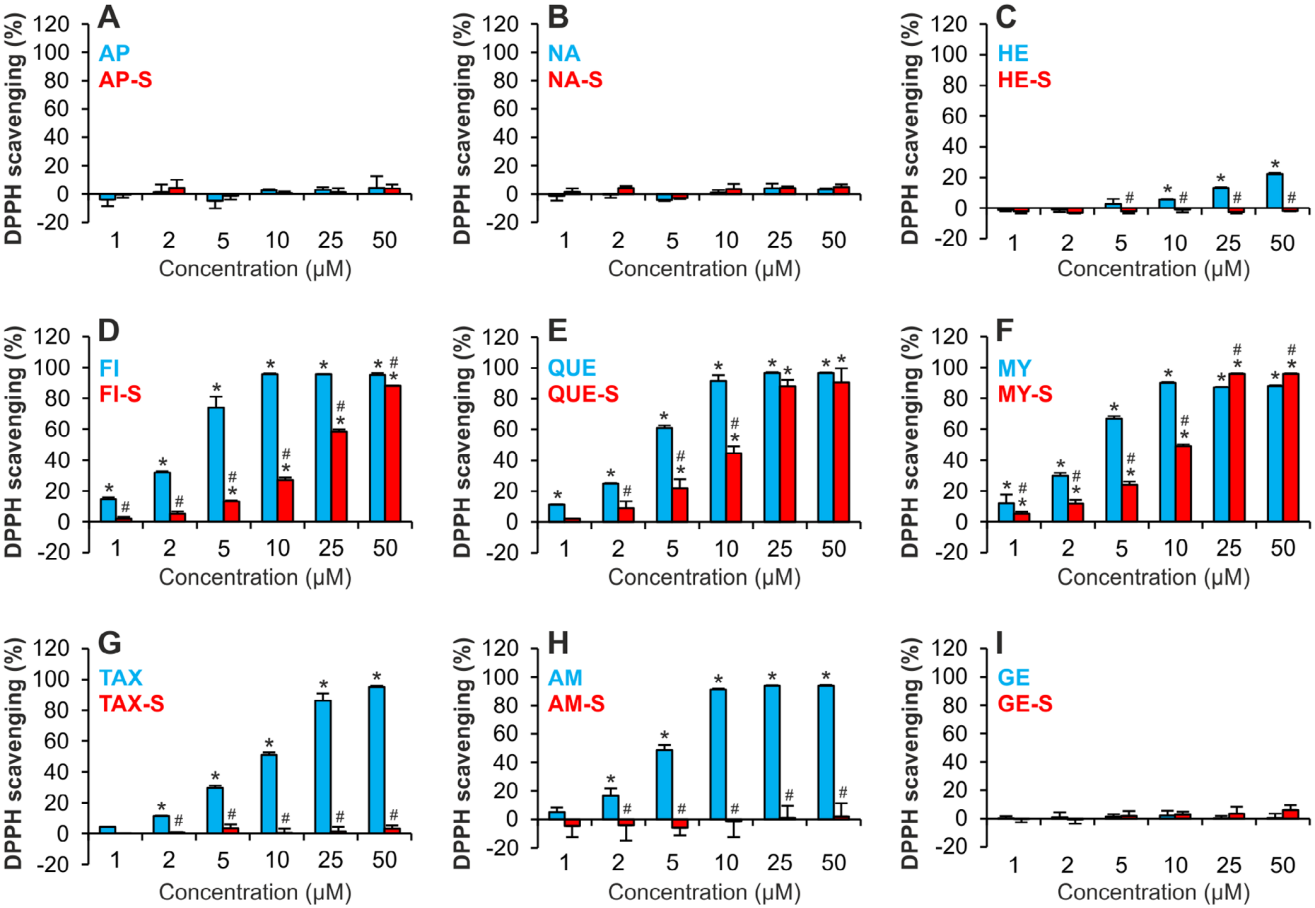
DPPH radical scavenging activity of tested flavonoids (blue) and their sulfates (red). (A) Apigenin; (B) naringenin; (C) hesperetin; (D) fisetin; (E) quercetin; (F) myricetin; (G) taxifolin; (H) ampelopsin; (I) genistein. Data are means ± SD of three experiments. *p < 0.05, significantly increased versus the control value (i.e., 0% at flavonoid concentration of 0 μM, not shown in the graphs). #*p* < 0.05, significantly different from the value for the respective nonsulfated flavonoid.

Antiradical activity was assessed using the DPPH assay, which monitors electron and/or hydrogen donation (Xie and Schaich 2014). After 30 min, apigenin, naringenin, genistein, and their sulfates showed negligible DPPH scavenging over the 1–50 μM range (Figure 4). In contrast, six nonsulfated flavonoids were active, with potency decreasing in the order fisetin ≈ myricetin > quercetin > ampelopsin > taxifolin >> hesperetin (Table 4). Flavonols and, to a lesser extent, flavanonols were the most effective scavengers (half‐maximal effective concentrations (EC_50_) 3.4–9.7 μM), highlighting the importance of a catechol or pyrogallol B ring and the 2,3‐double bond (Prochazkova et al. 2011).

Sulfation significantly reduced the antiradical activity. Fisetin‐3′‐*O*‐sulfate, quercetin‐3′‐*O*‐sulfate, and myricetin‐3′‐O‐sulfate retained DPPH scavenging ability but with significantly higher EC_50_ values than the parent flavonols (Table 3). The differences in the antiradical effect of sulfated and nonsulfated fisetin, quercetin, and myricetin were particularly apparent at medium concentrations of the tested compounds (e.g., 5 and 10 μM), but at the highest concentration tested (50 μM) the extent of DPPH scavenging reached 88%–97% for both sulfated and nonsulfated flavonols (Figure 4D–F), indicating that sulfation reduced the potency but not the maximal efficacy. In contrast, sulfation of taxifolin, ampelopsin, and hesperetin completely abolished the DPPH scavenging effect. These findings, consistent with previous reports (Roubalova et al. 2015), indicate that preservation of antiradical activity in sulfated flavonoids depends on the presence of the 2,3‐double bond and/or the position of sulfation.

**TABLE 3:** EC_50_ values for DPPH radical scavenging activity of tested flavonoids.

Compound	EC_50_ (μM)^a^	Sulfate	EC_50_ (μM)^a^
Apigenin	> 50	Apigenin‐4′‐*O*‐sulfate	> 50
Naringenin	> 50	Naringenin‐4′‐*O*‐sulfate	> 50
Hesperetin	> 50	Hesperetin‐3′‐*O*‐sulfate	> 50
Fisetin	3.4 ± 0.3	Fisetin‐3′‐*O*‐sulfate	16.9 ± 0.2*
Quercetin	4.2 ± 0.3	Quercetin‐3′‐*O*‐sulfate	10.3 ± 0.7*
Myricetin	3.4 ± 0.1	Myricetin‐3′‐*O*‐sulfate	10.5 ± 0.3*
Taxifolin	9.7 ± 1.1	Taxifolin‐4′‐*O*‐sulfate	> 50
Ampelopsin	5.1 ± 0.3	Ampelopsin‐4′‐*O*‐sulfate	> 50
Genistein	> 50	Genistein‐4′‐*O*‐sulfate	> 50

^a^ Data, calculated using OriginPro software, are means ± SD of three experiments. Values stated as > 50 indicate that a 50% scavenging effect was not reached at concentrations up to 50 μM.

* *p* < 0.05, significantly increased compared to the value for the respective parent flavonoid.

### 3.5 Cytotoxicity of Tested Flavonoids Toward RAW264.7 Cells

**FIGURE 5:**
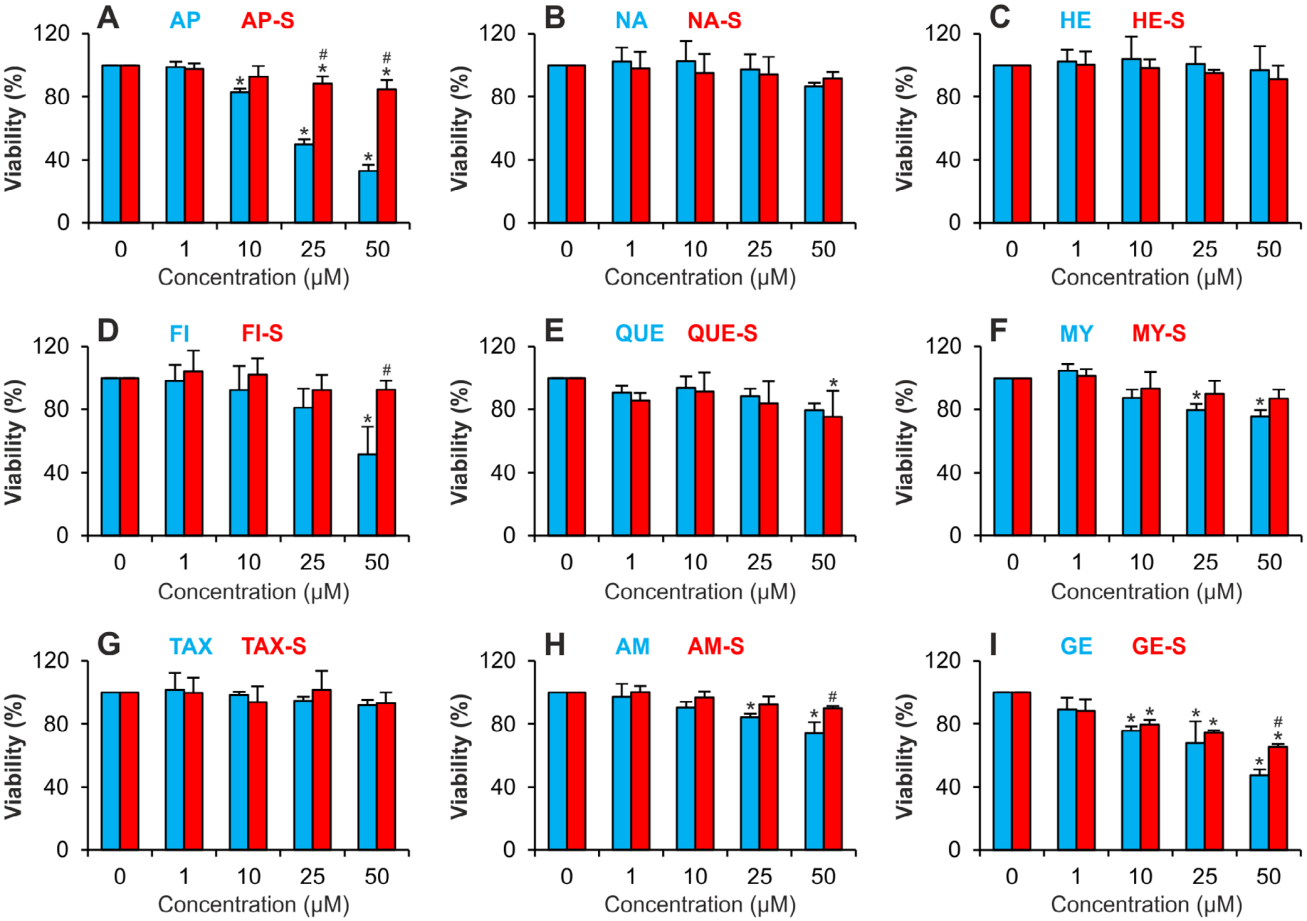
Effect of tested flavonoids (blue) and their sulfates (red) on the viability of RAW264.7 cells. Cells were treated in serum‐free medium with 0.1% DMSO (control) or 1–50 μM (A) apigenin, (B) naringenin, (C) hesperetin, (D) fisetin, (E) quercetin, (F) myricetin, (G) taxifolin, (H) ampelopsin or (I) genistein. The percentage of viable cells was determined by MTT assay. Data are means ± SD of three experiments. *p < 0.05, significantly decreased versus control. #*p* < 0.05, significantly increased versus the respective nonsulfated flavonoid.

Cytotoxicity of the tested flavonoids was assessed in RAW264.7 macrophages using the MTT assay after 24 h exposure to 1–50 μM compounds under serum‐free conditions. Naringenin, hesperetin, taxifolin, and their sulfates showed no or only minor cytotoxicity, with cell viability remaining at 86%–97% at 50 μM (Figure 5). In contrast, ampelopsin, myricetin, and quercetin moderately reduced viability to 74%–79%. Quercetin‐3′‐*O*‐sulfate exhibited cytotoxicity comparable to quercetin, whereas sulfation of ampelopsin and myricetin partially attenuated their cytotoxic effects. The strongest cytotoxicity was observed for apigenin, genistein, and fisetin, which decreased cell viability to 33%, 48%, and 52%, respectively, at 50 μM. Their sulfated derivatives were substantially less cytotoxic, with apigenin‐4′‐*O*‐sulfate, genistein‐4′‐*O*‐sulfate, and fisetin‐3′‐*O*‐sulfate maintaining cell viability at 85%, 66%, and 93%, respectively (Figure 5). Overall, these data indicate that B‐ring sulfation can markedly reduce flavonoid cytotoxicity. The most cytotoxic compounds were characterized by the presence of a 2,3‐double bond and a lower number of hydroxyl groups, typically three or four.

### 3.6 Effect of Tested Flavonoids on the Nrf2 Pathway in RAW264.7 Cells

The ability of the tested compounds to activate the NRF2 pathway, a key regulator of cellular redox homeostasis, was examined in RAW264.7 macrophages treated for 4 h with 50 μM flavonoids in serum‐free medium. According to the MTT assay, cell viability remained above 95% for all compounds except fisetin, which reduced viability to 88% (Figure S17A). The expression of NRF2 pathway components was analyzed at the mRNA and/or protein levels focusing on NRF2 (NFE2L2), its repressor KEAP1, and the NRF2‐regulated enzymes heme oxygenase‐1 (HO‐1/HMOX1) and NAD(P)H:quinone oxidoreductase 1 (NQO1) (Hayes and Dinkova‐Kostova 2014).

**FIGURE 6:**
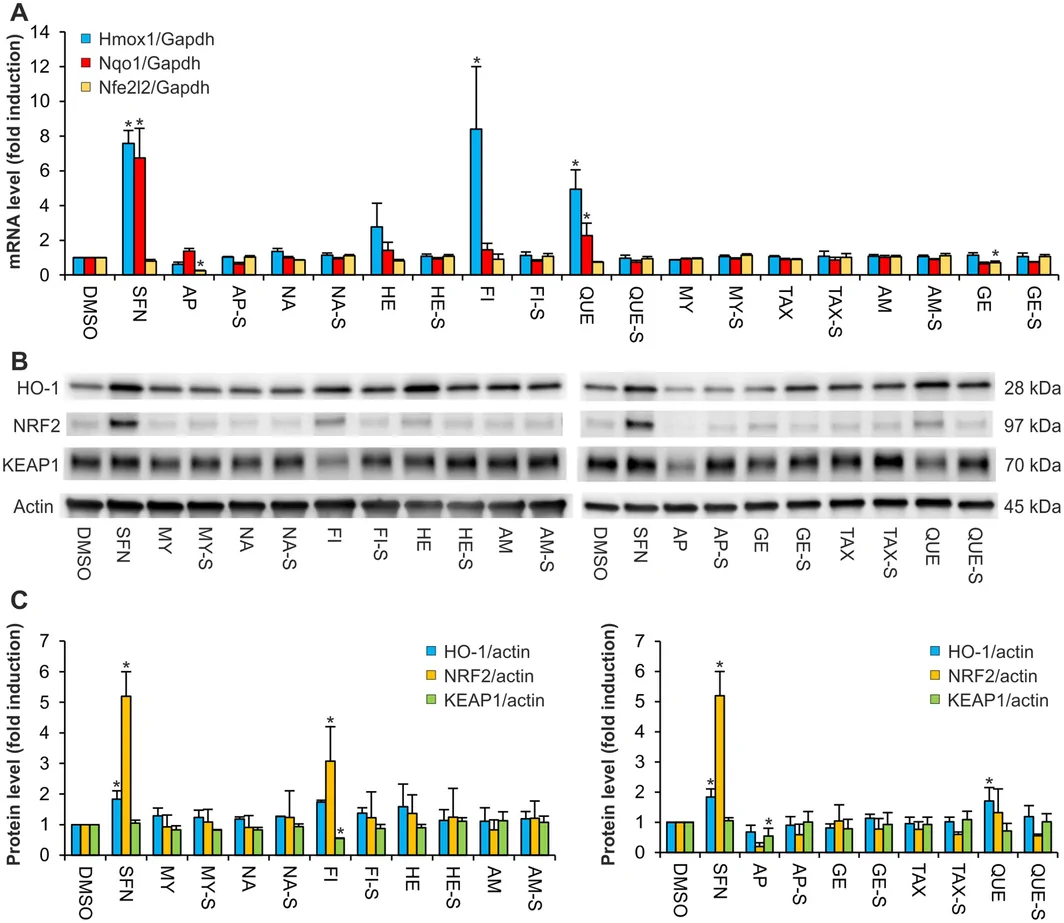
Effect of tested flavonoids and their sulfates on NRF2‐dependent gene expression in RAW264.7 cells. Cells were treated for 4 h with 0.1% DMSO (control), 5 μM sulforaphane (SFN, positive control), or 50 μM flavonoids (for their abbreviations, see Figure 1). (A) The levels of Hmox1, Nqo1, and Nfe2l2 (Nrf2) mRNA were determined by PCR and normalized to Gapdh mRNA. Data are means ± SD of three experiments. *p < 0.05, significantly different from control. (B, C) Protein levels of HO‐1, NRF2, KEAP1, and actin were analyzed in whole cell lysates by Western blotting (15 μg/lane). (B) Representative immunoblots. Uncropped membranes are shown in Figure S18. (C) Relative HO‐1, NRF2, and KEAP1 band intensities, normalized to actin. Data are means ± SD of three experiments. *p < 0.05, significantly different from control.

Sulforaphane (5 μM), used as a positive control (Ahn et al. 2010), induced a strong upregulation of Hmox1 and Nqo1 mRNA (7.6‐fold and 6.7‐fold, respectively), while slightly decreasing Nfe2l2 mRNA (Figure 6A). Among the tested flavonoids, fisetin and quercetin significantly induced Hmox1 mRNA (8.4‐fold and 4.9‐fold, respectively), accompanied by moderate increases in Nqo1 mRNA. Hesperetin caused minor increases in Hmox1 and Nqo1 expression (2.8‐fold and 1.4‐fold, respectively). In contrast, apigenin, naringenin, myricetin, taxifolin, ampelopsin, genistein, and all flavonoid sulfates failed to induce Hmox1 or Nqo1 mRNA and in some cases reduced their expression. Notably, apigenin and genistein significantly downregulated Nfe2l2 mRNA to 0.2‐fold and 0.7‐fold of control, respectively (Figure 6A). At the protein level, sulforaphane markedly increased NRF2 and HO‐1 levels without affecting KEAP1 (Figure 6B,C). Fisetin and quercetin also upregulated HO‐1 protein, accompanied by accumulation of NRF2 and decrease in KEAP1 protein levels. Other compounds caused only minor or inconsistent changes, with apigenin uniquely decreasing NRF2, KEAP1, and HO‐1 protein levels (Figure 6B,C).

Overall, the efficient activation of the NRF2 pathway was restricted to fisetin and quercetin. These findings are consistent with previous studies on fisetin (Zhang et al. 2019) and quercetin (Roubalova et al. 2016; Tanigawa et al. 2007; Vrba et al. 2013), while the inhibitory effect of apigenin and/or the inactivity of genistein differed from earlier reports (Huang et al. 2013; Zhai et al. 2013). Under present experimental conditions, the sulfate‐conjugated flavonoids did not induce the NRF2‐dependent expression in RAW264.7 cells.

### 3.7 Effect of Tested Flavonoids on COX‐2 and iNOS Expression in LPS‐Stimulated RAW264.7 Cells

**FIGURE 7:**
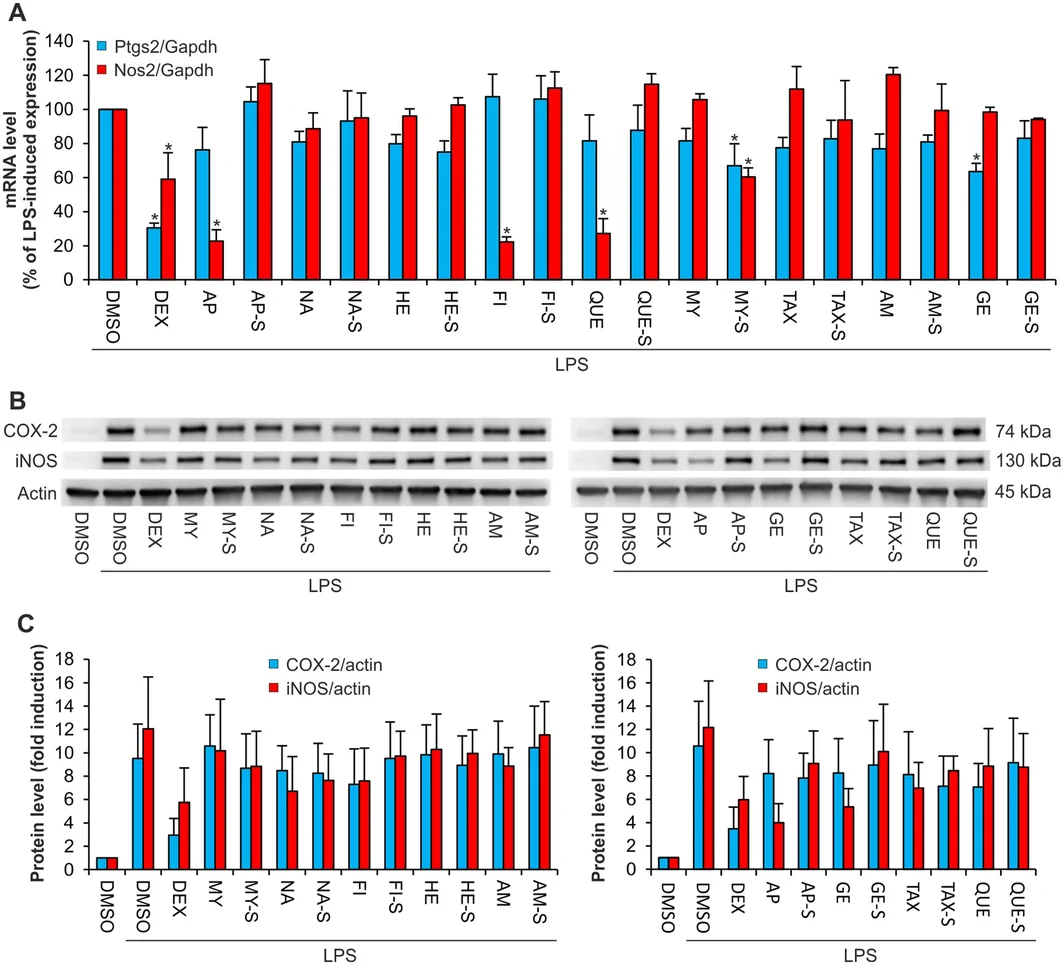
Effect of tested flavonoids on the expression of COX‐2 and iNOS in LPS‐stimulated RAW264.7 cells. Cells were pretreated for 2 h with 0.1% DMSO (control), 1 μM dexamethasone (DEX, positive control), or 10 μM flavonoids (for their abbreviations, see Figure 1), and then co‐treated for additional (A) 4 h or (B, C) 22 h with tested compounds and 0.1 μg/mL LPS. (A) The levels of Ptgs2 (Cox2) and Nos2 mRNA were determined by PCR and normalized to Gapdh mRNA. Data are means ± SD of three experiments. *p < 0.05, significantly different from LPS‐treated control. LPS alone induced (40 ± 9)‐fold and (256 ± 71)‐fold increases in the levels of Ptgs2 and Nos2 mRNA, respectively, compared to untreated control (not shown in the graph). (B, C) Protein levels of COX‐2, iNOS, and actin were analyzed in whole cell lysates by Western blotting (15 μg/lane). (B) Representative immunoblots. Uncropped membranes are shown in Figure S19. (C) Relative COX‐2 and iNOS band intensities, normalized to actin. Data are means ± SD of three experiments.

The effects of the tested compounds on inflammatory signaling were evaluated in LPS‐stimulated RAW264.7 cells, a well‐established model of NF‐κB–mediated induction of cyclooxygenase‐2 (COX‐2/PTGS2) and inducible nitric oxide synthase (iNOS/NOS2) (Gutschow et al. 2013; Zhong et al. 2022). Cells were pretreated with flavonoids (10 μM) or dexamethasone (1 μM) (Chuang et al. 2017) and subsequently cotreated with LPS (0.1 μg/mL). Cell viability remained ≥ 85% under all treatment conditions (Figure S17B). LPS induced (40 ± 9)‐fold and (256 ± 71)‐fold increases (*p* < 0.05) in the levels of Ptgs2 and Nos2 mRNA, respectively, and this effect was significantly suppressed by dexamethasone (Figure 7A).

Of the tested flavonoids, genistein and myricetin‐3′‐*O*‐sulfate significantly reduced LPS‐induced Ptgs2 mRNA expression. A significant suppression of Nos2 mRNA was observed for fisetin, apigenin, quercetin, and myricetin‐3′‐*O*‐sulfate (Figure 7A). At the protein level, changes in expression were not statistically significant due to high SD values. However, dexamethasone markedly decreased both COX‐2 and iNOS expression stimulated by LPS (Figure 7B,C). LPS‐induced COX‐2 protein levels were partially reduced by most tested compounds, with the strongest effects observed for fisetin, quercetin, and taxifolin‐4′‐*O*‐sulfate. LPS‐induced iNOS levels were decreased by all flavonoids, with apigenin and genistein showing effects comparable to dexamethasone (Figure 7B,C).

Collectively, these data demonstrate that several flavonoids, including selected sulfated derivatives, attenuate COX‐2 and iNOS expression in LPS‐stimulated RAW264.7 macrophages. Consistent with previously published results, apigenin, genistein (Liang et al. 1999), fisetin, and quercetin (Zhong et al. 2022) showed relevant effects. In contrast, flavonoid sulfates such as taxifolin‐4′‐*O*‐sulfate and myricetin‐3′‐*O*‐sulfate were generally weak modulators; nonetheless, myricetin‐3′‐*O*‐sulfate was more effective than the parent compound.

LPS‐induced expression of *Ptgs2* and *Nos2* genes is primarily mediated through Toll‐like receptor 4 (TLR4) signaling, which activates multiple downstream pathways including NF‐κB, activator protein‐1 (AP‐1), and mitogen‐activated protein kinase (MAPK) cascades (Park and Lee 2013). Our data demonstrated that several flavonoids and their sulfated derivatives attenuated the expression of LPS‐induced inflammatory markers at the mRNA and/or protein levels in RAW264.7 cells; nonetheless, the specific signaling pathways involved remain to be determined. Given the lower cellular uptake and higher protein‐binding capacity of sulfated flavonoids (Kaci et al. 2023; Roubalova et al. 2015), we may speculate that the effect of flavonoid sulfates on the LPS‐induced response could involve interactions with extracellular proteins or cell surface receptors. The sulfate group is ionized at physiological pH, and it markedly reduces passive membrane permeability; consequently, intracellular concentrations of the tested sulfates were likely lower than the nominal medium concentration. It should nevertheless be noted that circulating flavonoid Phase II conjugates, including quercetin‐3′‐*O*‐sulfate, are substrates of organic anion transporters (OATs), which mediate their uptake into tissues (Wong et al. 2012). Moreover, flavonoid conjugates are known to affect various human cell types at concentrations as low as 0.1 μM (Williamson 2025), and they can also be deconjugated by sulfatases and β‐glucuronidases at sites of inflammation, locally releasing the more permeable aglycones (Williamson 2025). The finding that several flavonoid sulfates attenuated COX‐2 and iNOS expression despite their limited passive permeability may therefore suggest that their potency may be underestimated in the present cell model.

In addition, differential effects on COX‐2 and iNOS expression deserve note. For instance, fisetin, quercetin, and apigenin significantly reduced Nos2 mRNA but not Ptgs2 mRNA levels, while myricetin‐3′‐*O*‐sulfate suppressed the expression of both genes. At the protein level, effects were similarly non‐parallel, for instance, with apigenin and genistein, which apparently decreased iNOS protein levels stronger than those of COX‐2. Such divergence may reflect differential regulation of the two genes. In addition to NF‐κB, the expression of COX‐2 depends on the transcription factors CREB (the cyclic‐AMP response element binding protein) and C/EBP (the CCAAT‐enhancer binding protein) (Tsatsanis et al. 2006), while the expression of iNOS may additionally involve IRF‐1 (interferon regulatory factor 1) (Cinelli et al. 2020). The ability of sulfated flavonoids to modulate COX‐2 and/or iNOS expression without affecting the NRF2 pathway indicates that the suppression of LPS‐induced inflammatory markers is independent of the KEAP1/NRF2 axis, and it likely involves multiple, partially divergent mechanisms. We suggest that the elucidation of the molecular basis for the differential effect of flavonoids on the expression of COX‐2 and iNOS should be the focus of future research.

Thus, a limitation of the present study is that NF‐κB pathway activation was not directly assessed; no direct indicators such as p65 (RelA) phosphorylation and nuclear translocation, IκBα degradation, or NF‐κB‐dependent reporter activity were measured. Consequently, our conclusions are restricted to the modulation of LPS‐induced inflammatory markers, and the specific signaling step(s) affected by the tested compounds remain to be identified. Direct measurement of NF‐κB nuclear translocation and upstream signaling events will be particularly informative for the sulfated flavonoids: given that the ionized sulfate group markedly reduces passive membrane permeability, such data could help discriminate whether the sulfates act extracellularly (e.g., by interfering with LPS or the Toll‐like receptor 4 and its co‐receptors myeloid differentiation factor 2 and cluster of differentiation 14 (TLR4/MD‐2/CD14) receptor complex) (Park and Lee 2013), or after their cellular entry. Experiments focused on the effect of the active flavonoids on the NF‐κB and other signaling pathways together with quantification of intracellular flavonoid concentrations are therefore planned as part of a follow‐up study.

### 3.8 Involvement of Human Sulfotransferases (SULTs) in Flavonoid Biotransformation

Finally, involvement of human sulfotransferases in flavonoid biotransformation was examined to evaluate whether the tested sulfates can be formed by human enzymes. Parent flavonoids were incubated with recombinant SULTs 1A1*1, 1A1*2, 1A2, 1A3, 1B1, 1C2, 1C4, 1E1, and 2A1, and sulfation products were analyzed by LC–MS. Taxifolin was omitted from this part of the study, as data on its sulfation were published previously (Vrba et al. 2020). All compounds were efficiently sulfated by multiple SULT enzymes (Table 4), with the number of sulfation products ranging from two for hesperetin to six for ampelopsin. Based on the minimal percentage of residual parent compounds, their susceptibility to the enzymatic sulfation decreased in the following order: ampelopsin > apigenin > genistein ≈ naringenin ≈ hesperetin > quercetin ≈ fisetin > myricetin. Analyses revealed that monosulfated metabolites were only produced, while di‐ or trisulfated products were not found with any of the tested compounds under the present experimental conditions. Of the tested enzymes, SULT1C4 showed the highest overall activity with the tested flavonoids, except for hesperetin, which was preferentially sulfated by SULTs 1A3 and 1E1; these two enzymes also contributed substantially to the sulfation of the other flavonoids. Additionally, SULTs 1A1*1 and 1A1*2 were relevant for the sulfation of ampelopsin, naringenin, and hesperetin, SULT1A2 sulfated mainly hesperetin and naringenin, SULT1B1 sulfated mainly ampelopsin and hesperetin, and SULT1C2 showed some activity toward apigenin and naringenin. In contrast, SULT2A1 had negligible activity toward the tested compounds. Each tested flavonoid sulfate was produced by one or more SULT enzymes and authenticated by retention times, MS spectra, and spiking experiments. The maximal yields of individual sulfates ranged from 5% to 44%, with SULT1E1 generating the highest amounts of most sulfates, except for myricetin‐3′‐O‐sulfate, which was mainly formed by SULTs 1C4 and 1A3 (Table 4). Taxifolin‐4′‐*O*‐sulfate was previously identified as a minor product of SULTs 1E1 and 1A2 (Vrba et al. 2020).

**TABLE 4:** Sulfation of tested flavonoids by human sulfotransferases (SULTs).

Compound^a^	m/z^b^	Retention time	Semiquantitative percentage^c^								
	[M – H]^−^	(min)	1A1*1	1A1*2	1A2	1A3	1B1	1C2	1C4	1E1	2A1
*Apigenin (parent)*	269.0452	3.90	97.3	99.1	92.3	64.8	94.6	95.3	40.0	51.0	100.0
4′‐*O*‐Sulfate (1)	349.0011	5.11	2.6	0.0	0.0	0.0	0.0	0.0	0.0	33.7	0.0
Sulfate (2)	349.0011	5.26	0.0	0.1	0.1	0.0	5.4	4.7	60.0	15.3	0.0
Sulfate (3)	349.0011	5.32	0.1	0.8	7.6	35.2	0.0	0.0	0.0	0.0	0.0
*Naringenin (parent)*	271.0604	3.82	88.9	91.1	80.2	55.3	97.9	95.3	51.0	64.7	99.9
Sulfate (1)	351.0173	3.42	0.2	0.2	0.0	0.0	0.0	0.0	0.0	2.6	0.0
4′‐*O*‐Sulfate (2)	351.0173	4.84	5.2	4.0	1.1	0.0	0.0	0.0	0.7	22.5	0.0
Sulfate (3)	351.0173	5.08	5.7	4.7	18.7	44.7	2.1	4.7	48.3	10.2	0.1
*Hesperetin (parent)*	301.0705	3.96	89.9	91.0	76.8	52.2	79.8	99.1	79.4	54.9	99.8
3′‐*O*‐Sulfate (1)	381.0284	4.98	9.8	8.8	21.8	33.9	17.6	0.0	0.1	43.8	0.0
Sulfate (2)	381.0284	5.42	0.3	0.2	1.4	13.9	2.6	0.9	20.5	1.3	0.2
*Fisetin (parent)*	285.0407	3.03	99.0	99.4	98.0	71.2	94.0	99.8	59.6	63.6	100.0
Sulfate (1)	364.9967	3.74	0.0	0.0	0.6	8.2	0.1	0.2	12.2	3.0	0.0
3′‐*O*‐Sulfate (2)	364.9967	4.15	0.0	0.0	0.8	1.3	0.0	0.0	0.5	19.5	0.0
Sulfate (3)	364.9967	4.28	1.0	0.6	0.6	19.3	5.9	0.0	27.7	13.9	0.0
*Quercetin (parent)*	301.0358	3.48	99.1	99.4	97.5	67.1	96.3	99.9	57.6	75.9	100.0
Sulfate (1)	380.9909	3.35	0.0	0.0	0.0	0.0	0.0	0.0	0.0	0.1	0.0
Sulfate (2)	380.9909	4.43	0.0	0.0	0.9	1.1	0.0	0.1	15.8	0.7	0.0
3′‐*O*‐Sulfate (3)	380.9909	5.11	0.0	0.0	0.2	0.3	0.0	0.0	0.0	19.3	0.0
Sulfate (4)	380.9909	5.32	0.9	0.6	1.4	31.5	3.7	0.0	26.6	4.0	0.0
*Myricetin (parent)*	317.0303	3.00	99.3	99.3	97.4	82.3	94.7	99.8	67.9	82.4	100.0
Sulfate (1)	396.9869	3.59	0.0	0.0	1.7	0.9	0.0	0.2	0.7	0.3	0.0
3′‐*O*‐Sulfate (2)	396.9869	4.31	0.7	0.7	0.4	14.8	5.3	0.0	31.4	1.8	0.0
Sulfate (3)	396.9869	5.39	0.0	0.0	0.5	2.0	0.0	0.0	0.0	15.5	0.0
*Ampelopsin (parent)*	319.0454	2.17	45.1	77.0	91.5	30.0	71.0	99.8	19.7	84.5	100.0
Sulfate (1)	399.0020	2.02	0.1	0.0	0.0	0.0	0.0	0.0	0.0	1.2	0.0
Sulfate (2)	399.0020	2.41	0.1	0.0	4.0	1.7	0.0	0.2	2.4	1.2	0.0
Sulfate (3)	399.0020	2.63	0.0	0.0	0.5	0.6	0.0	0.0	0.3	0.2	0.0
Sulfate (4)	399.0020	3.15	52.0	23.0	4.0	67.7	26.3	0.0	69.3	4.7	0.0
Sulfate (5)	399.0020	3.24	2.7	0.0	0.0	0.0	2.7	0.0	8.3	3.1	0.0
4′‐*O*‐Sulfate (6)	399.0020	3.96	0.0	0.0	0.0	0.0	0.0	0.0	0.0	5.1	0.0
*Genistein (parent)*	269.0447	3.87	97.1	97.7	93.6	94.7	99.5	99.9	50.6	67.4	99.7
Sulfate (1)	349.0017	3.80	0.0	0.0	0.0	0.1	0.0	0.0	0.0	0.0	0.0
4′‐*O*‐Sulfate (2)	349.0017	4.84	0.1	0.1	0.2	4.9	0.4	0.0	0.6	16.3	0.0
Sulfate (3)	349.0017	5.08	2.8	2.2	6.2	0.3	0.1	0.1	48.8	16.3	0.3

*Note:* The tested flavonoids (50 μM) were incubated for 30 min with individual SULTs and then analyzed by LC–MS. Taxifolin was omitted from this part of the study (previously published in (Vrba et al. 2020)).

^a^ The numbers in brackets indicate elution order of the detected monosulfated products according to their retention times. The sulfation products with a defined position of the sulfate group (i.e., 3′‐*O*‐ and 4′‐*O*‐sulfates) were identified using the tested flavonoid sulfates as internal standards.

^b^ The m/z values were calculated using Metabolynx v4.1 software.

^c^ The semiquantitative percentage values, presented as means from three experiments, were calculated based on the peak area values. The sum of a given parent compound and its sulfation products equals 100%.

Our results demonstrate that the tested flavonoids can be sulfated by human SULTs 1A1, 1A2, 1A3, 1B1, 1C2, 1C4, and 1E1. SULT1A2 is probably not expressed at the protein level in human tissues (Nowell et al. 2005), and both SULT1C enzymes are expressed mainly during the fetal period (Coughtrie 2016). In contrast, SULTs 1A1, 1A3, 1B1, and 1E1 are found in the small intestine and, except for SULT1A3, in the liver (Coughtrie 2016; Riches et al. 2009). The involvement of the intestinal and hepatic human sulfotransferases in the formation of the tested flavonoid sulfates suggests that they can be considered as plausible human metabolites.

### 3.9 Integrated Structure–Property‐Activity Relationship (SAR/SPAR) Analysis

**FIGURE 8:**
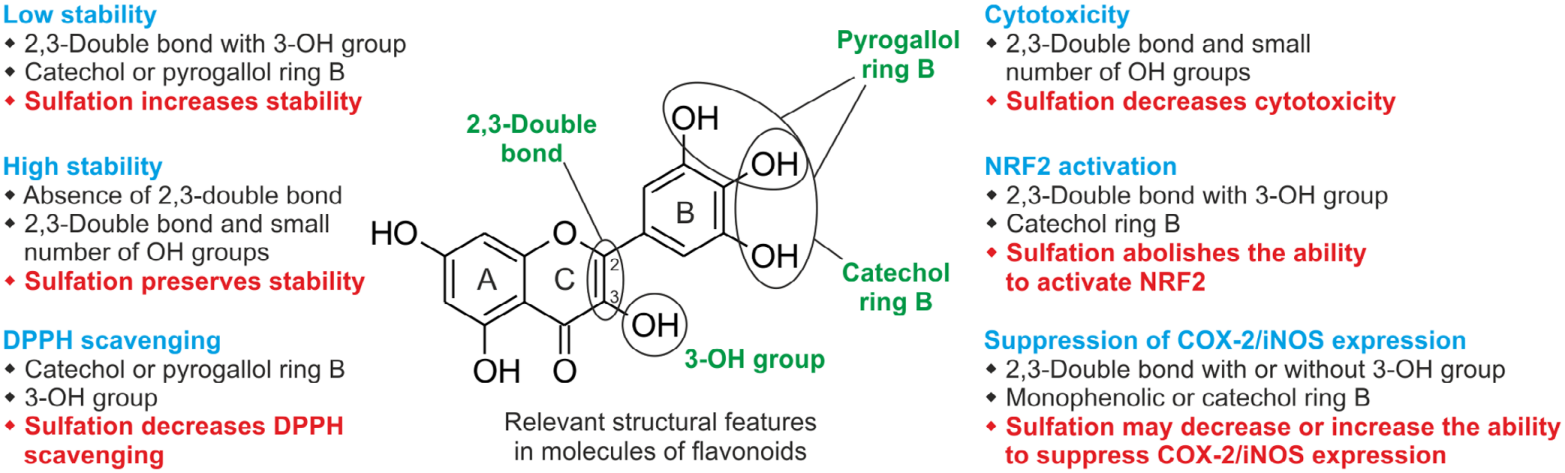
Summary of structure–property‐activity relationships of tested flavonoids. An example of flavonoid structure (myricetin) and its structural features (described in green) are shown in the middle of the scheme. On the left and right side of the scheme, the investigated properties/activities are stated in blue, the structural features consistently contributing to the given properties/activities are stated in black, and the effect of the ring B sufation is described in red.

When the five endpoints examined in this study, i.e., stability, DPPH antiradical activity, cytotoxicity, NRF2 pathway activation, and anti‐inflammatory response were considered together, several consistent structure–property‐activity relationships emerged as summarized in Figure 8. The presence of the 2,3‐double bond and 3‐OH group in conjunction with a catechol or pyrogallol ring B appeared to be the single most influential structural determinant, conferring to flavonols, particularly fisetin and quercetin, a high antiradical activity, NRF2 activation capacity, and suppressing effect on Nos2 mRNA, but at the cost of a marked instability at the physiological pH. The flavonol myricetin failed to exert a relevant effect on the NRF2 pathway and LPS‐induced inflammatory response, presumably due to its high lability associated with a high number of hydroxyl groups and/or pyrogallol ring B. Flavanones and flavanonols, which lack the 2,3‐double bond, were stable and largely inactive toward DPPH and NRF2, yet they retained the ability to modulate inflammatory markers. Apigenin (flavone) and genistein (isoflavone) occupied a distinct SAR/SPAR space: they possess the 2,3‐double bond but lack the catechol/pyrogallol B‐ring substitution, resulting in a profile of a high stability, negligible antiradical activity, elevated cytotoxicity, and a potent suppression of iNOS protein expression.

SAR/SPAR analysis showed that the effect of sulfation varied according to the endpoint examined, rather than according to the parent structure. Most strikingly, the sulfation forbids the activation of the NRF2 pathway across all nine flavonoids tested, irrespective of their parent potency or sulfation position. This contrasted with the tested anti‐inflammatory activity, where the sulfation produced a spectrum of outcomes, including attenuation, retention, or even enhancement of the effect on LPS‐induced expression, indicating that the structural determinants of COX‐2/iNOS modulation are at least partially distinct from those governing NRF2 activation. The stronger anti‐inflammatory effect of myricetin‐3′‐*O*‐sulfate, compared to myricetin, could reflect the higher stability of the sulfated molecule. The sulfation, however, consistently stabilized all of the labile flavonoids, with the degree of stabilization depending on the parent structure. As expected, sulfated flavanonol ampelopsin was more stable than sulfated flavonols. For DPPH scavenging, the sulfation of hesperetin (flavanone), taxifolin, and ampelopsin (flavanonols) completely abolished the antiradical activity. In contrast, 3′‐*O*‐sulfation of fisetin, quercetin, and myricetin (flavonols) only partially decreased the antiradical effect, presumably due to the presence of the 2,3‐double bond and one or two residual hydroxyl groups on ring B in the sulfated flavonoids (Figure 8). These SAR/SPAR relationships underscore the importance of evaluating flavonoid metabolites on a case‐by‐case basis and provide a framework for predicting the biological consequences of metabolic sulfation across the flavonoid family.

## 4 Concluding Remarks

This study demonstrates that metabolic sulfation represents a critical determinant of flavonoid behavior under physiologically relevant conditions. Regioselective sulfation markedly enhances the stability of labile flavonoids while attenuating their antiradical capacity, cytotoxicity, and ability to activate the NRF2 pathway. At the same time, several flavonoid sulfates retain the capacity to modulate LPS‐induced inflammatory responses, indicating that sulfated metabolites are not biologically inert. Identification of human sulfotransferases responsible for their formation further supports the relevance of these compounds as plausible metabolites. From a nutritional perspective, these findings carry several important implications. First, the marked differences in stability between parent flavonoids and their sulfated conjugates indicate that the molecular species reaching systemic circulation and peripheral tissues after dietary flavonoid intake differ fundamentally from those present in food. Flavonols such as quercetin and fisetin, which are abundant in fruits and vegetables, are intrinsically unstable at the physiological pH, yet are stabilized by the B ring sulfation, suggesting that the sulfation preserves their potential to exert biological effects during systemic distribution. Conversely, the loss of the NRF2‐activating capacity upon sulfation demonstrates that metabolism does not merely attenuate activity but can qualitatively redirect the biological effects of dietary flavonoids. The retention of the anti‐inflammatory activity by several sulfated conjugates is particularly noteworthy. This supports the concept that circulating flavonoid metabolites, including monosulfates, are not simply inactive excretion products destined for elimination but active participants in the modulation of inflammatory response. Given that recent pharmacokinetic data demonstrate that quercetin monosulfates and hetero‐conjugates represent the predominant circulating species in humans following dietary intake (Sudaka et al. 2025), the ability of the sulfated metabolites to modulate COX‐2 and iNOS expression becomes relevant to understanding the epidemiological associations between flavonoid‐rich diets and reduced risk of chronic inflammatory diseases (Williamson 2017). The identification of specific human SULT enzymes capable of generating the tested flavonoid sulfates, including SULTs 1A1, 1A3, 1B1, and 1E1 expressed in the small intestine and liver (Coughtrie 2016; Riches et al. 2009), further confirms that these compounds are plausible circulating species after dietary intake.

Regarding the translational relevance of the concentrations employed, it should be noted that total circulating flavonoid conjugates can reach concentrations approaching 10 μM in human plasma after supplementation with appropriately formulated flavonoid preparations (Williamson 2025), supporting the physiological plausibility of the concentrations used herein for the anti‐inflammatory experiments. While the 50 μM concentration used to probe the NRF2 pathway activation is supraphysiological, it served to establish the maximal response capacity of each compound; the complete loss of NRF2 activation observed upon sulfation thus strongly suggests that sulfated metabolites would also be inactive at lower, physiologically attainable levels. Conversely, the retained or even enhanced anti‐inflammatory activity of certain flavonoid sulfates at 10 μM indicates that these metabolites may contribute to the modulation of inflammatory signaling at concentrations relevant to dietary intake.

Together, these findings highlight the necessity of including sulfated conjugates in mechanistic and nutritional studies of flavonoids and provide a framework for more accurate interpretation of their biological effects in vivo. A limitation of this study is that the effect on NF‐κB and other inflammatory signaling pathways was not directly investigated, and intracellular concentrations of the test compounds were not determined. Because sulfation reduces passive membrane permeability, the relative potency of sulfated versus parent flavonoids may be underestimated. Examination of the effect on the NF‐κB and other signaling pathways, together with quantification of cellular uptake, as well as assessment of sulfatase‐mediated deconjugation in inflamed tissues, will be important for refining structure–activity relationships in future work.

## Author Contributions

**Jiří Vrba:** conceptualization, investigation, writing – original draft, formal analysis, writing – review and editing, methodology, funding acquisition, supervision. **Veronika Zapletalová:** investigation, writing – review and editing. **Jakub Havlásek:** investigation, writing – review and editing. **Barbora Papoušková:** investigation, methodology, writing – review and editing, formal analysis, data curation. **Barbora Petránková:** investigation, writing – review and editing. **Helena Pelantová:** investigation, writing – review and editing, data curation. **Kateřina Valentová:** conceptualization, funding acquisition, writing – original draft, writing – review and editing, project administration, supervision.

## Funding

This work was supported by the Czech Science Foundation (23‐04654S) and by the Ministry of Education, Youth and Sports of the Czech Republic (CZ.02.01.01/00/22_008/0004597 and LUC25026).

## Disclosure

During manuscript revision, the authors used the WebUI at https://chat.ai.e‐infra.cz, specifically the kimi‐k2.6 and deepseek‐v4‐pro‐thinking models (accessed July 2026) to assist in refining discussion text. All generative artificial intelligence (AI)‐assisted outputs were critically reviewed, revised, and approved by the authors. No AI tools were used for experimental data generation or analysis.

## Supporting information


**Table S1:** Sulfation conditions and major sulfation products of apigenin, hesperetin, fisetin, and genistein.
**Figure S1:** HPLC chromatogram of apigenin 4′‐*O*‐sulfate.
**Figure S2:** ^1^H NMR spectrum of apigenin 4′‐*O*‐sulfate.
**Figure S3:** ^13^C NMR spectrum of apigenin 4′‐*O*‐sulfate.
**Table S2:** ^1^H and ^13^C NMR data of apigenin 4′‐*O*‐sulfate.
**Figure S4:** MS spectrum of apigenin 4′‐*O*‐sulfate.
**Figure S5:** HPLC chromatogram of fisetin‐3′‐*O*‐sulfate.
**Figure S6:** ^1^H NMR spectrum of fisetin‐3′‐*O*‐sulfate.
**Figure S7:** ^13^C NMR spectrum of fisetin‐3′‐*O*‐sulfate.
**Table S3:** ^1^H and ^13^C NMR data of fisetin‐3′‐*O*‐sulfate.
**Figure S8:** MS spectrum of fisetin‐3′‐*O*‐sulfate.
**Figure S9:** HPLC chromatogram of genistein 4′‐*O*‐sulfate.
**Figure S10:** ^1^H NMR spectrum of genistein 4′‐*O*‐sulfate.
**Figure S11:** ^13^C NMR spectrum of genistein 4′‐*O*‐sulfate.
**Table S4:** ^1^H and ^13^C NMR data of genistein 4′‐*O*‐sulfate.
**Figure S12:** MS spectrum of genistein 4′‐*O*‐sulfate.
**Figure S13:** HPLC chromatogram of hesperetin 3′‐*O*‐sulfate.
**Figure S14:** ^1^H NMR spectrum of hesperetin 3′‐*O*‐sulfate.
**Figure S15:** ^13^C NMR spectrum of hesperetin 3′‐*O*‐sulfate.
**Table S5:** ^1^H and ^13^C NMR data of hesperetin 3′‐*O*‐sulfate.
**Figure S16:** MS spectrum of hesperetin 3′‐*O*‐sulfate.
**Table S6:** Absorption spectral properties of flavonoids in phosphate buffer (pH 7.4).
**Figure S17:** Effect of flavonoids on the viability of RAW264.7 cells.
**Figure S18:** Uncropped membranes corresponding to Western blot analyses of proteins in RAW264.7 cells.
**Figure S19:** Uncropped membranes corresponding to Western blot analyses of proteins in RAW264.7 cells.

## Data Availability

The data that support the findings of this study are openly available in Zenodo at https://zenodo.org/, reference number https://doi.org/10.5281/zenodo.18604657.
