# Supplementary material for: Sulfation Rewrites the Biological Profile of Dietary Flavonoids: Implications for Stability, Redox Signaling, and Inflammation
Source: Food Sci Nutr. 2026 Sep 21;14(9):e72382. doi: 10.1002/fsn3.72382 (PMC13594397; doi:10.1002/fsn3.72382)
Supplement: Supplementary file 1 [file FSN3-14-e72382-s001.docx]

Supplementary Material

Sulfation Rewrites the Biological Profile of Dietary Flavonoids: Implications for Stability, Redox Signaling, and Inflammation

Jiří Vrba^1^, Veronika Zapletalová^1^, Jakub Havlásek^1^, Barbora Papoušková^2^, Barbora Petránková^3,4^, Helena Pelantová^5^, Kateřina Valentová^3,^*

^1^ Department of Medical Chemistry and Biochemistry, Faculty of Medicine and Dentistry, Palacký University, Olomouc, Czechia

^2^ Department of Analytical Chemistry, Faculty of Science, Palacký University, Olomouc, Czechia

^3^ Institute of Microbiology of the Czech Academy of Sciences, Laboratory of Biotransformation, Prague, Czechia

^4^ Department of Genetics and Microbiology, Faculty of Science, Charles University, Prague, Czechia

^5^ Institute of Microbiology of the Czech Academy of Sciences, Laboratory of Molecular Structure Characterization, Prague, Czechia

* Corresponding author:

*E-mail:* [kata.valentova@email.cz](mailto:kata.valentova@email.cz) (dr. Kateřina Valentová)

**Table of Contents**

Supplementary Methods	3

Flavonoid Sulfation	3

**Table S1**. Sulfation Conditions and Major Sulfation Products of Apigenin, Hesperetin, Fisetin, and Genistein	3

HPLC Analysis of Synthesized Flavonoid Sulfates	3

NMR Analysis of Synthesized Flavonoid Sulfates	4

MS Analysis of Synthesized Flavonoid Sulfates	4

LC-MS Analysis of Flavonoid Sulfates Produced by Human Sulfotransferases	4

Supplementary Results	5

**Fig. S1.** HPLC chromatogram of apigenin 4′-*O*-sulfate.	5

**Fig. S2.** ^1^H NMR spectrum of apigenin 4′-*O*-sulfate	5

**Fig. S3.** ^13^C NMR spectrum of apigenin 4′-*O*-sulfate	6

**Table S2.** ^1^H and ^13^C NMR data of apigenin 4′-*O*-sulfate	6

**Fig. S4.** MS spectrum of apigenin 4′-*O*-sulfate.	7

**Fig. S5.** HPLC chromatogram of fisetin-3′-*O*-sulfate.	7

**Fig. S6.** ^1^H NMR spectrum of fisetin-3′-*O*-sulfate	8

**Fig. S7.** ^13^C NMR spectrum of fisetin-3′-*O*-sulfate	8

**Table S3.** ^1^H and ^13^C NMR data of fisetin-3′-*O*-sulfate	9

**Fig. S8.** MS spectrum of fisetin-3′-*O*-sulfate.	9

**Fig. S9.** HPLC chromatogram of genistein 4′-*O*-sulfate.	10

**Fig. S10.** ^1^H NMR spectrum of genistein 4′-*O*-sulfate	10

**Fig. S11.** ^13^C NMR spectrum of genistein 4′-*O*-sulfate	11

**Table S4.** ^1^H and ^13^C NMR data of genistein 4′-*O*-sulfate	11

**Fig. S12.** MS spectrum of genistein 4′-*O*-sulfate.	12

**Fig. S13.** HPLC chromatogram of hesperetin 3′-*O*-sulfate.	12

**Fig. S14.** ^1^H NMR spectrum of hesperetin 3′-*O*-sulfate	13

**Fig. S15.** ^13^C NMR spectrum of hesperetin 3′-*O*-sulfate	13

**Table S5.** ^1^H and ^13^C NMR data of hesperetin 3′-*O*-sulfate	14

**Fig. S16.** MS spectrum of hesperetin 3′-*O*-sulfate.	14

**Table S6.** Absorption spectral properties of flavonoids in phosphate buffer (pH 7.4).	15

**Fig. S17.** Effect of flavonoids on the viability of RAW264.7 cells.	16

**Fig. S18.** Uncropped membranes corresponding to Western blot analyses of proteins in RAW264.7 cells.	17

**Fig. S19.** Uncropped membranes corresponding to Western blot analyses of proteins in RAW264.7 cells.	18

# Supplementary Methods

## Flavonoid Sulfation

Each parent flavonoid was dissolved in 5 mL acetone, followed by the addition of *p*-NPS in Tris–glycine buffer (24 mL, 100 mM, pH 8.9) and AST enzyme (2 mL crude lysate). The reaction mixture was incubated under an inert atmosphere (Ar) in a shaking incubator at 30 °C and 200 rpm in a flask with a septum-sealed lid. Reaction progress was monitored by TLC, using a mobile phase of ethyl acetate/methanol/formic acid (4:1:0.01), with detection by UV light and spraying with H_2_SO_4_ in ethanol. Upon complete conversion, the reaction was stopped by heating to 95 °C. Acetone was removed under reduced pressure, the pH was adjusted to 7.5–7.7 with formic acid, and the mixture was extracted with ethyl acetate (3 × 50 mL) to remove *p*-nitrophenol and any unreacted substrate. The aqueous layer, containing the sulfated product, was evaporated, dissolved in 2–5 mL of 80% aqueous methanol, and purified by chromatography on a Sephadex LH-20 column (GE Healthcare Bio-Sciences, Uppsala, Sweden; 30 g dry weight, 3 cm i.d.) packed and equilibrated with the same solvent. Elution was performed over 2–4 days at 20 °C and a flow rate of 0.16 mL/min; 4-mL fractions were collected and analyzed by TLC as described above. Fractions containing the target product were combined, evaporated at 45 °C, lyophilized, and characterized by HPLC, MS, and NMR. Details of the individual sulfation reactions of apigenin, fisetin, genistein, and hesperetin are provided in Table 1.

### **Table S1**. Sulfation Conditions and Major Sulfation Products of Apigenin, Hesperetin, Fisetin, and Genistein

| Substrate | m (mg) | m*_p_*_-NPS_ (mg) | Reaction time (h) | Product |
| --- | --- | --- | --- | --- |
| Apigenin | 203 | 162 (1 eq) | 5.5 | Apigenin-4′-*O*-sulfate |
| Fisetin | 206 | 153 (1 eq) | 5.5 | Fisetin-3′-*O*-sulfate |
| Genistein | 100 | 114 (1.2 eq) | 5 | Genistein-4′-*O*-sulfate |
| Hesperetin | 202 | 145 (1 eq) | 5.5 | Hesperetin-3′-*O*-sulfate |

## HPLC Analysis of Synthesized Flavonoid Sulfates

Data from HPLC analyses were obtained using the Shimadzu Prominence LC analytical system, which included the Shimadzu CBM-20A system controller, Shimadzu LC-20AD binary HPLC pump, Shimadzu CTO-10AS column oven, Shimadzu SIL-20ACHT cooling autosampler, and Shimadzu SPD-20MA diode array detector (Shimadzu, Kyoto, Japan). Separation of flavonoids and their sulfates was performed on a Kinetex PFP column (150 × 4.6 mm, 5 µm) with a PFP guard column (4 × 3 mm, 5 µm) from Phenomenex (Torrance, CA, USA). Mobile phase A was 10 mM ammonium acetate in water containing 0.1% (*v/v*) formic acid (pH 3.8), and mobile phase B was 100% methanol. The gradient elution was as follows: 0–20 min 40–72% B, 20–21 min 72–40% B, and 21–24 min 40% B for column equilibration. The flow rate was 0.6 mL/min, the column oven temperature was 45 °C, and PDA detection was set at 200–400 nm. The absorption maximum wavelength of each respective compound was extracted.

## NMR Analysis of Synthesized Flavonoid Sulfates

NMR data were acquired on Bruker Avance III 600 MHz and 400 MHz spectrometers (Bruker BioSpin, Rheinstetten, Germany) in DMSO-*d*_6_ at 30 °C. Standard ^1^H NMR, ^13^C NMR, gCOSY, ^1^H-^13^C gHSQC, and ^1^H-^13^C gHMBC experiments were performed using the manufacturer's software TopSpin 3.5. Individual spin systems, assigned by COSY and HSQC experiments, were combined using information from the HMBC experiment.

## MS Analysis of Synthesized Flavonoid Sulfates

Samples were dissolved in methanol/acetonitrile (1:1, *v/v*, methanol was added to increase the solubility) and injected (1 µL) into the mobile phase of acetonitrile (300 µL/min) using a 50-µL loop. The spray, capillary, tube lens voltage, and capillary temperature were set to 3.5 kV, −16 V, −120 V, and 250 °C, respectively. A single quadrupole mass spectrometer Shimadzu LC-MS-2020 (Shimadzu) equipped with electrospray ionization was used.

## LC-MS Analysis of Flavonoid Sulfates Produced by Human Sulfotransferases

The parent flavonoids were sulfated by recombinant human sulfotransferases as described in section 2.10 of the main text. For analysis by liquid chromatography-mass spectrometry (LC-MS), the samples were thawed, mixed 1:1 (*v/v*) with methanol containing 5% (*v/v*) acetic acid, centrifuged for 5 min at 12,000 × *g* and 4 °C, and the supernatants were analyzed. Analyses were performed using an Acquity UPLC I-Class system (Waters, Milford, MA, USA) equipped with a solvent manager, sample manager, and column manager with a Kinetex 1.7 µm EVO C18 column (100 × 2.1 mm i.d.; Phenomenex, Torrance, CA, USA). The mobile phase consisted of 0.1% (*v/v*) formic acid in water (A) and 0.1% (*v/v*) formic acid in acetonitrile (B). The column oven was maintained at 40 °C, and the elution was performed at a flow rate of 0.55 mL/min with the following linear gradient: 0–8 min 0–85% B, 8–9 min 85% B, and 9–9.5 min 85–100% B, followed by a return to initial conditions and equilibration at 0% B. Samples were maintained in the autosampler at 10 °C, and the injection volume was 3 µL. The electrospray ionization source of a Synapt G2-S Mass Spectrometer (Waters) was operated in negative mode with the following settings: capillary voltage 2.25 kV; sampling cone 35 V; source temperature 120 °C; desolvation temperature 300 °C; cone gas flow 25 L/h; and desolvation gas flow 600 L/h. Data in the range of 50–1200 Da were collected with a scan time of 0.2 s, and a sodium formate solution in acetonitrile was used for instrument calibration. Data were automatically processed and corrected for mass error during acquisition using leucine enkephalin as an external reference. Data were obtained using two interleaved scan functions (MS^E^ experiments), enabling simultaneous acquisition of both low-collision-energy (4 eV) and high-collision-energy (15-30 eV) mass spectra. Post-acquisition analysis was performed using Metabolynx v4.1 software (Waters). MS data with ppm ≥ 5 were not considered.

# Supplementary Results

### **Fig. S1.** HPLC chromatogram of apigenin 4′-*O*-sulfate.

### **Fig. S2.** ^1^H NMR spectrum of apigenin 4′-*O*-sulfate

(600.23 MHz for ^1^H, DMSO-*d*_6_, 30 °C).

### **Fig. S3.** ^13^C NMR spectrum of apigenin 4′-*O*-sulfate

(150.93 MHz for ^13^C, DMSO-*d*_6_, 30 °C).

### **Table S2.** ^1^H and ^13^C NMR data of apigenin 4′-*O*-sulfate

(600.23 MHz for ^1^H, 150.93 MHz for ^13^C, DMSO-*d*_6_, 30 °C).

| Atom | δ_C_ | m. | δ_H_ | n_H_ | m. | *J* [Hz] | δ_C_^apigenin^ | δ_C_ - δ_C_^apigenin^ |
| --- | --- | --- | --- | --- | --- | --- | --- | --- |
| 2 | 162.93 | S | - | 0 | - | - | 163.65 | -0.72 |
| 3 | 103.86 | D | 6.824 | 3 | s | - | 102.76 | 1.10 |
| 4 | 181.44 | S | - | 0 | - | - | 181.64 | -0.20 |
| 5 | 161.32 | S | - | 0 | - | - | 161.36 | -0.04 |
| 6 | 99.17 | D | 6.149 | 1 | d | 2.1 | 98.73 | 0.44 |
| 7 | 165.62 | S | - | 0 | - | - | 164.03 | 1.59 |
| 8 | 94.17 | D | 6.448 | 1 | d | 2.1 | 93.86 | 0.31 |
| 9 | 157.41 | S | - | 0 | - | - | 157.21 | 0.20 |
| 10 | 103.17 | S | - | 0 | - | - | 103.61 | -0.44 |
| 1’ | 124.77 | S | - | 0 | - | - | 121.09 | 3.68 |
| 2’6’ | 127.42^x^ | D | 7.988 | 2 | m | Σ*J* = 8.9 | 128.36^x^ | -0.94 |
| 3’5 | 119.96^x^ | D | 7.343 | 2 | m | Σ*J* = 8.9 | 115.86^x^ | 4.10 |
| 4’ | 156.77 | S | - | 0 | - | - | 161.06 | -4.29 |
| 5-OH | - | - | 12.867 | 1 | s | - | - | - |
| 7-OH | - | - | n.d. | - | - | - | - | - |

^x^ ,2C; n.d., not detected.

### **Fig. S4.** MS spectrum of apigenin 4′-*O*-sulfate.

### **Fig. S5.** HPLC chromatogram of fisetin-3′-*O*-sulfate.

### **Fig. S6.** ^1^H NMR spectrum of fisetin-3′-*O*-sulfate

(600.23 MHz for ^1^H, DMSO-*d*_6_, 30 °C).

### **Fig. S7.** ^13^C NMR spectrum of fisetin-3′-*O*-sulfate

(150.93 MHz for ^13^C, DMSO-*d*_6_, 30 °C).

### **Table S3.** ^1^H and ^13^C NMR data of fisetin-3′-*O*-sulfate

(600.23 MHz for ^1^H, 150.93 MHz for ^13^C, DMSO‑*d*_6_, 30 °C).

| Atom | δ_C_ | m. | δ_H_ | n_H_ | m. | *J* [Hz] | δ_C_^fisetin^ | δ_C_ - δ_C_^fisetin^ |
| --- | --- | --- | --- | --- | --- | --- | --- | --- |
| 2 | 144.18 | S | - | 0 | - | - | 144.95 | -0.77 |
| 3 | 137.27 | S | - | 0 | - | - | 137.06 | 0.21 |
| 4 | 171.87 | S | - | 0 | - | - | 171.85 | 0.02 |
| 5 | 126.28 | D | 7.914 | 1 | m | - | 126.34 | -0.06 |
| 6 | 114.76 | D | 6.885 | 1 | m | - | 114.55 | 0.21 |
| 7 | 162.62 | S | - | 0 | - | - | 162.14 | 0.48 |
| 8 | 101.75 | D | 6.90^H^ | 1 | m | - | 101.72 | 0.03 |
| 9 | 156.25 | S | - | 0 | - | - | 156.19 | 0.06 |
| 10 | 113.91 | S | - | 0 | - | - | 114.12 | -0.21 |
| 1’ | 122.72 | S | - | 0 | - | - | 122.41 | 0.31 |
| 2’ | 122.40 | D | 8.016 | 1 | m | - | 114.87 | 7.53 |
| 3’ | 140.69 | S | - | 0 | - | - | 144.93 | -4.24 |
| 4’ | 150.70 | D | - | 0 | - | - | 147.14 | 3.56 |
| 5’ | 117.10 | D | 6.980 | 1 | d | 8.6 | 115.47 | 1.63 |
| 6’ | 124.58 | D | 7.854 | 1 | dd | 8.6, 2.2 | 119.53 | 5.05 |

^H^ , HSQC readout; hydroxyl signals were not assigned.

### **Fig. S8.** MS spectrum of fisetin-3′-*O*-sulfate.

### **Fig. S9.** HPLC chromatogram of genistein 4′-*O*-sulfate.

### **Fig. S10.** ^1^H NMR spectrum of genistein 4′-*O*-sulfate

(399.67 MHz for 1H, DMSO-d6, 30 °C).

### **Fig. S11.** ^13^C NMR spectrum of genistein 4′-*O*-sulfate

(100.50 MHz for ^13^C, DMSO-*d*_6_, 30 °C).

### **Table S4.** ^1^H and ^13^C NMR data of genistein 4′-*O*-sulfate

(399.67 MHz for ^1^H, 100.50 MHz for ^13^C, DMSO-*d*_6_, 30 °C).

| Atom | δ_C_ | m. | δ_H_ | n_H_ | m. | *J* [Hz] | δ_C_^genistein^ | δ_C_ - δ_C_^genistein^ |
| --- | --- | --- | --- | --- | --- | --- | --- | --- |
| 2 | 154.07 | D | 8.331 | 1 | s | - | 153.70 | 0.37 |
| 3 | 121.83 | S | - | 0 | - | - | 122.28 | -0.45 |
| 4 | 179.62 | S | - | 0 | - | - | 180.15 | -0.53 |
| 5 | 161.84 | S | - | 0 | - | - | 161.99 | -0.15 |
| 6 | 99.35 | D | 6.173 | 1 | d | 2.1 | 98.94 | 0.41 |
| 7 | 165.90 | S | - | 0 | - | - | 164.21 | 1.69 |
| 8 | 93.87 | D | 6.327 | 1 | d | 2.1 | 93.62 | 0.25 |
| 9 | 157.62 | S | - | 0 | - | - | 157.54 | 0.08 |
| 10 | 103.74 | S | - | 0 | - | - | 104.46 | -0.72 |
| 1’ | 125.38 | S | - | 0 | - | - | 121.26 | 4.12 |
| 2’6’ | 129.34^x^ | D | 7.455 | 2 | m | Σ*J* = 8.8 | 130.11 | -0.77 |
| 3’5 | 120.00^x^ | D | 7.218 | 2 | m | Σ*J* = 8.8 | 115.05 | 4.95 |
| 4’ | 153.40 | S | - | 0 | - | - | 157.39 | -3.99 |
| 5-OH | - | - | 12.896 | 1 | br s | - | - | - |
| 7-OH | - | - | n.d. | - | - | - | - | - |

^x^ , 2C; n.d., not detected.

### **Fig. S12.** MS spectrum of genistein 4′-*O*-sulfate.

### **Fig. S13.** HPLC chromatogram of hesperetin 3′-*O*-sulfate.

### **Fig. S14.** ^1^H NMR spectrum of hesperetin 3′-*O*-sulfate

(600.23 MHz for ^1^H, DMSO-*d*_6_, 30 °C).

### **Fig. S15.** ^13^C NMR spectrum of hesperetin 3′-*O*-sulfate

(150.93 MHz for ^13^C, DMSO-*d*_6_, 30 °C).

### **Table S5.** ^1^H and ^13^C NMR data of hesperetin 3′-*O*-sulfate

(600.23 MHz for ^1^H, 150.93 MHz for ^13^C, DMSO-*d*_6_, 30 °C).

| Atom | δ_C_ | m. | δ_H_ | n_H_ | m. | *J* [Hz] | δ_C_^hesperetin^ | δ_C_ - δ_C_^hesp.^ |
| --- | --- | --- | --- | --- | --- | --- | --- | --- |
| 2 | 78.12 | D | 5.451 | 1 | dd | 12.7, 3.1 | 78.10 | 0.02 |
| 3 | 42.02 | T | 3.159 | 1 | dd | 17.1, 12.7 | 41.97 | 0.05 |
|  |  |  | 2.684 | 1 | dd | 17.1, 3.1 | - | - |
| 4 | 195.72 | S | - | 0 | - | - | 196.04 | -0.32 |
| 5 | 163.37 | S | - | 0 | - | - | 163.37 | 0.00 |
| 6 | 95.83 | D | 5.860 | 1 | d | 2.2 | 95.70 | 0.13 |
| 7 | 166.9454 | S | - | 0 | - | - | 166.54 | 0.40 |
| 8 | 95.00 | D | 5.888 | 1 | d | 2.2 | 94.89 | 0.11 |
| 9 | 162.66 | S | - | 0 | - | - | 162.69 | -0.03 |
| 10 | 101.54 | S | - | 0 | - | - | 101.72 | -0.18 |
| 1’ | 130.21 | S | - | 0 | - | - | 131.09 | -0.88 |
| 2’ | 119.70 | D | 7.591 | 1 | d | 2.3 | 113.98 | 5.72 |
| 3’ | 142.51 | S | - | 0 | - | - | 146.40 | -3.89 |
| 4’ | 150.79 | D | - | 0 | - | - | 147.80 | 2.99 |
| 5’ | 112.47 | D | 6.994 | 1 | d | 8.5 | 111.97 | 0.50 |
| 6’ | 121.65 | D | 7.135 | 1 | dd | 8.5, 2.3 | 117.84 | 3.81 |
| 4’-OMe | 55.71 | Q | 3.758 | 3 | s | - | 55.61 | 0.1 |

Hydroxyl signals were not assigned

### **Fig. S16.** MS spectrum of hesperetin 3′-*O*-sulfate.

### **Table S6.** Absorption spectral properties of flavonoids in phosphate buffer (pH 7.4).

| Compound | λ in nm (ε in M^-1^ cm^-1^) |
| --- | --- |
| Apigenin | 270 (21800), 352 (16150) |
| Apigenin 4′-*O*-sulfate | 271 (30200), 342 (12750) |
| Naringenin | 322 (23500) |
| Naringenin 4′-*O*-sulfate | 322 (17650) |
| Hesperetin | 322 (19950) |
| Hesperetin 3′-*O*-sulfate | 322 (15350) |
| Fisetin | 249 (17900), 321 (14050), 369 (20950) |
| Fisetin 3′-*O*-sulfate | 257 (7350), 321 (6600), 362 (9250) |
| Quercetin | 266 (14900), 320 (8150), 378 (14250) |
| Quercetin 3′-*O*-sulfate | 269 (17450), 306 (9650), 374 (14850) |
| Myricetin | 252 (15600), 329 (12400), 377 (13250) |
| Myricetin 3′-*O*-sulfate | 262 (14300), 378 (13250) |
| Taxifolin | 324 (17000) |
| Taxifolin 4′-*O*-sulfate | 325 (21500) |
| Ampelopsin | 325 (23800) |
| Ampelopsin 4′-*O*-sulfate | 325 (25200) |
| Genistein | 264 (28850) |
| Genistein 4′-*O*-sulfate | 266 (23400) |

The data were obtained from absorption spectra of 10 µM flavonoids in 50 mM phosphate buffer (pH 7.4), recorded at the time point of 0 h. For details, see section 2.4 in the main text. Wavelengths of absorption maxima (λ) were determined with an accuracy of 1 nm, and molar absorption coefficients (ε) were calculated according to Lambert–Beer's law.

### **Fig. S17.** Effect of flavonoids on the viability of RAW264.7 cells.

(**A**) Cells were treated for 4 h in serum-free medium with 0.1% (*v/v*) DMSO (control) or 50 µM apigenin (AP), apigenin 4′-*O*-sulfate (AP-S), naringenin (NA), naringenin 4′-*O*-sulfate (NA-S), hesperetin (HE), hesperetin 3′-*O*-sulfate (HE-S), fisetin (FI), fisetin 3′-*O*-sulfate (FI-S), quercetin (QUE), quercetin 3′-*O*-sulfate (QUE-S), myricetin (MY), myricetin 3′-*O*-sulfate (MY-S), taxifolin (TAX), taxifolin 4′-*O*-sulfate (TAX-S), ampelopsin (AM), ampelopsin 4′-*O*-sulfate (AM-S), genistein (GE), or genistein 4′-*O*-sulfate (GE-S). (**B**) Cells were pretreated for 2 h in serum-containing medium with 0.1% DMSO (controls) or 10 µM flavonoids (see above), and then the cells were incubated for additional 22 h with the tested compounds in the presence or absence of 0.1 µg/mL lipopolysaccharide (LPS). After treatments, the percentage of viable cells was determined by MTT assay. Data are means ± SD of three experiments. No significant changes were found.

### **Fig. S18.** Uncropped membranes corresponding to Western blot analyses of proteins in RAW264.7 cells.

For detailed results, see Fig. 6B in the main text.

### **Fig. S19.** Uncropped membranes corresponding to Western blot analyses of proteins in RAW264.7 cells.

For detailed results, see Fig. 7B in the main text.
